# Empagliflozin Ameliorates Preeclampsia and Reduces Postpartum Susceptibility to Adriamycin in a Mouse Model Induced by Angiotensin Receptor Agonistic Autoantibodies

**DOI:** 10.3389/fphar.2022.826792

**Published:** 2022-03-23

**Authors:** Ruonan Zhai, Yuan Liu, Jiahao Tong, Ying Yu, Lin Yang, Yong Gu, Jianying Niu

**Affiliations:** ^1^ Department of Nephrology, Shanghai Fifth People’s Hospital, Fudan University, Shanghai, China; ^2^ Department of Nephrology, Huashan Hospital, Fudan University, Shanghai, China

**Keywords:** preeclampsia, empagliflozin, autoantibody against angiotensin II type 1 receptor, podocyte, oxidative stress

## Abstract

Preeclampsia (PE) is the leading cause of maternal and perinatal morbidity and mortality and also is a risk factor for cardiovascular and kidney disease later in life. PE is associated with oversecretion of autoantibodies against angiotensin II type 1 receptor (AT1-AA) by the placenta into the maternal circulation. Here, we sought to determine the therapeutic value of the sodium-glucose co-transporter 2 (SGLT2) inhibitor empagliflozin (EMPA) in mice with AT1-AA-induced preeclampsia. Pregnant mice were injected with AT1-AA at gestation day (GD) 13 and treated daily with EMPA until GD 19, at which point some of the maternal mice were sacrificed and assessed. The other maternal mice were labored on time and challenged with adriamycin (ADR) at 12 weeks postpartum; their offspring were assessed for fetal outcomes. We showed that EMPA treatment significantly relieved high systolic blood pressure and proteinuria and ameliorated kidney injury in PE mice without affecting fetal outcomes. EMPA also ameliorated podocyte injury and oxidative stress, reduced the expression of SGLT2 and activated the AMPK/SIRT1 signaling pathway *in vivo* and *in vitro*. Remarkably, EMPA treatment during pregnancy reduced ADR-induced kidney and podocyte injury postpartum. These findings suggest that EMPA could be a potential pharmacological agent for PE.

## Introduction

Preeclampsia (PE) is characterized by unprecedented onset of hypertension (blood pressure ≥140/90 mm Hg) along with evidence of maternal organ failure (Brown et al., 2018). PE is estimated to affect 5–7% of all pregnant women and is responsible for over 70,000 maternal deaths and 500,000 fetal deaths worldwide every year (Hogan et al., 2010). Even worse, women who experience PE are at increased risk (five times) for the development of end-stage renal disease in the long term (Covella et al., 2019; Khashan et al., 2019). According to current research and knowledge, PE results from generalized endothelial damage, which is caused by placental syncytiotrophoblast factors released into the maternal circulation (Burton et al*.*, 2019). Studies have found that podocyte excretion occurs in patients with PE (Garovic et al., 2007; Craici et al., 2013), suggesting that the preeclamptic kidney sustains not only endothelial damage but also podocyte damage. The podocyte line, the external surface of the glomerular basement membrane (GBM), consists of a cellular body, major processes and foot processes (FPs) (Greka & Mundel, 2012). The slit diaphragm (SD) bridges the filtration slits between opposing podocyte FPs and establishes the final barrier to urinary protein loss (Mundel & Kriz, 1995; Greka & Mundel, 2012). The SDs between Fps are made up of a set of molecules involved in cell adhesion and tight cell-to-cell junctions, including nephrin, podocin and CD2AP. Defects to these proteins lead to proteinuria (Kawachi & Fukusumi, 2020). Studies have found increased concentrations of nephrin in the urine of PE patients, which correlate positively with PE severity (Wang et al., 2015).

Despite the detrimental effects that PE can cause to both mothers and fetuses, the only definitive treatment for PE is delivery of the fetus (Phipps et al., 2019). Thus, it is urgent to develop effective pharmacological therapeutics to improve immediate and long-term prognosis. Sodium-glucose co-transporter 2 (SGLT2) inhibitors were originally developed as antihyperglycemic agents and increase urinary glucose excretion by inhibiting SGLT2 co-transporters located in the proximal convoluted tubule of the nephron (Basile, 2011). Recently, large randomized controlled trials have demonstrated both renal and cardiovascular protection in patients with or without type 2 diabetes (Perkovic et al., 2019; Heerspink et al., 2020; Packer et al., 2020). Since SGLT2 inhibitors can reduce blood pressure and proteinuria (Georgianos & Agarwal, 2019; Mosenzon et al., 2019), the typical clinical manifestations of preeclampsia. We hypothesized that SGLT2 has a protective effect on preeclampsia. SGLT2 inhibitors induce a shift to a “fasting state”, which in turn upregulates the expression of the energy deprivation sensors sirtuin-1 (SIRT1) and adenosine monophosphate-activated protein kinase (AMPK) (Packer, 2020b). This is part of the mechanism by which SGLT2 inhibitors reverse the development of metabolic syndrome (Kim et al., 2016).

During PE, placental ischemia is associated with oversecretion of autoantibodies against angiotensin II type 1 receptor (AT1-AA) into the maternal circulation. AT1-AA binds to the AT1 receptor (AT1R) and plays an agonist-like role (Lumbers et al., 2019). By activating AT1R, AT1-AA also stimulates downstream pathways, increases the production of circulating endothelin-1 and sFlt-1, and increases the levels of oxidative stress, which further leads to hypertension, maternal organ damage and fetal death (Campbell et al., 2018). Here, we applied an AT1-AA-induced PE model in C57BL/6 mice, which caused an increase in hypertension and kidney damage. Importantly, we demonstrate the successful use of this model to test the efficacy of the oral SGLT2 inhibitor empagliflozin (EMPA) to reverse the clinical characteristics of PE and reduce postpartum susceptibility to adriamycin (ADR).

## Material and Methods

### Reagents and Antibodies

Empagliflozin was purchased from MedChemExpress (MCE, CAS No.: 864,070-44-0, United States). Losartan potassium was purchased from Merck Pharmaceuticals Co. Ltd. (Merck Sharp & Dohme, Australia). Anti-synaptopodin (ab259976), anti-NPHS2 (ab50339), anti-AMPK alpha 1 (phospho T183) + AMPK alpha 2 (phospho T172) (ab133448) and anti-SOD2/MnSOD (ab68155) antibodies were purchased from Abcam Biotechnology (Abcam, England). Anti-SGLT-2 (D-6) (sc-393350) and anti-WT1 (F-6) (sc-7385) antibodies were purchased from Santa Cruz Biotechnology, Inc. (Santa Cruz, United States). Rabbit anti-NPHS1 polyclonal antibody (abs136679) was purchased from Absin Biochemical Company (Absin, Shanghai, China). Goat anti-AMPKα1 polyclonal antibody (AF3197) was purchased from R&D Systems (R&D, United States). Rabbit anti-FOXO1 (2800S) and mouse anti-β-actin (3700S) were purchased from Cell Signaling Technology (CST, United States). TRITC Phalloidin (40734ES75) was obtained from Yeasen Biotechnology, Inc. (Yeasen, Shanghai, China). The Cell Counting Kit-8 (CCK-8) was obtained from Dojindo Molecular Technologies, Inc. (Dojindo, Japan). The PrimeScriptTM RT Master Mix (Perfect Real Time) (RR036A) and TB Green Premix Ex TaqTM (Tli RNaseH Plus) (RR420A) were purchased from TaKaRa Biotechnology, Inc. (TaKaRa, Japan). Reactive oxygen species (ROS) assay kits (S0033) and doxorubicin hydrochloride (ADR) (ST1285) were obtained from Beyotime Biotechnology, Inc. (Beyotime, China).

### Preparation of AT1-AA

AT1-AA-negative guinea pigs were immunized with the functional second-loop epitope peptide of human AT1R (AT1RECII, sequence IHRNVFFIENTNITVCAFHYESQNSTL) for 10 weeks. Sera were collected from the guinea pigs, modified ELISA was used to detect the AT1-AA titers and AT1-AA-IgG was purified and used in the following animal and cell experiments.

### Animal Experiments

**FIGURE 1:**
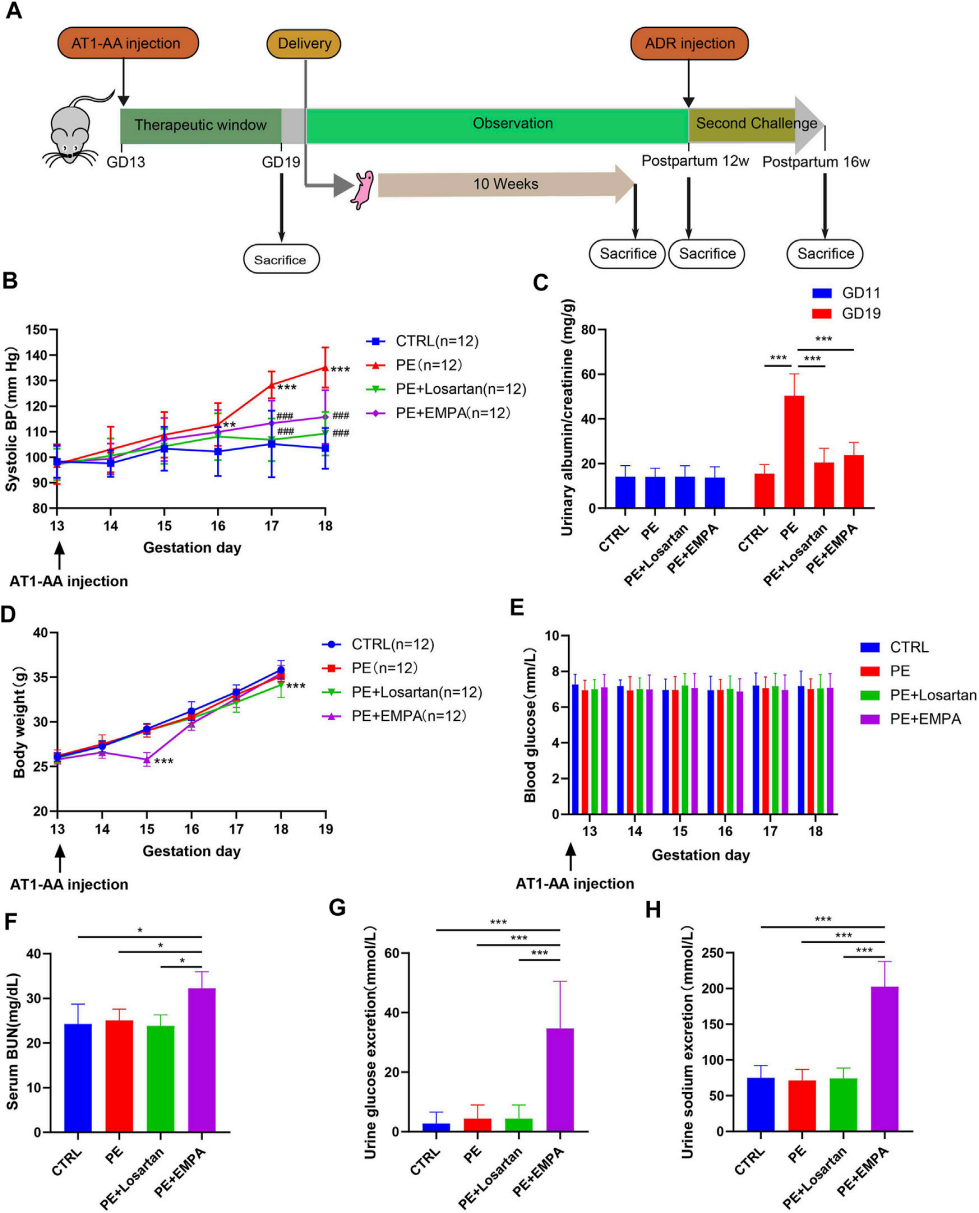
EMPA reduced SBP and ACR and induced glycosuria and natriuresis in an AT1-AA-infused mouse model. **(A)** Schematic illustration of animal experiments. **(B)** Systolic blood pressure (SBP) recorded from GD13 to GD18 of control or PE mice treated with EMPA or losartan (*n* = 12). **(C)** Urinary albumin to creatinine ratio (ACR) on GD11 and GD19 in control and PE mice (*n* = 12). **(D,E)** Mouse body weight and blood glucose recorded from GD13 to GD18 in each group (*n* = 12). Mouse serum BUN **(F)** (*n* = 3), urine glucose excretion **(G)** (*n* = 12) and urine sodium excretion **(H)** (*n* = 12) of each group. Data are expressed as the mean ± SEM and were analyzed by ANOVA with LSD post hoc test. *p < 0.05, **p < 0.01, ***p < 0.001.

All animal procedures (no. A2020062) were approved by the Institutional Animal Care and Use Committee at Shanghai Jiaotong University (Shanghai, China). 8-week-old female C57BL/6 mice and male C57BL/6 mice were purchased from Beijing Vital River Laboratory Animal Technology Co., Ltd. (Beijing, China), and all mice were housed in a temperature- and humidity-controlled room with a 12:12 h light-dark cycle. They were fed standard laboratory animal feed and had free access to water. Virgin female C57BL/6 mice were mated with male C57BL/6 mice after they were allowed to acclimate for 2 weeks. Gestation day (GD) 1 was defined by the presence of a white or faint yellow vaginal plug the following morning. Pregnant mice were randomly divided into the control group, PE group, PE+losartan group or PE+EMPA group. In addition, we added the losartan group and EMPA group to test the effect of losartan and EMPA on the quantity and survival rate of their offspring. The experimental PE model was induced by injection of AT1-AA (20 μg/g body weight) in 200 μL of sterile saline into the tail vein on GD 13 according to previous reports (Zhou et al*.*, 2008). Normal pregnant control mice were injected with an equal volume of saline. Losartan group and PE+losartan group mice were orally administered losartan (10 mg/kg/d) from GD13 to GD19 (Zhang et al., 2017). EMPA group and PE+EMPA group mice were orally administered EMPA (30 mg/kg/d) from GD13 to GD19; The dose of EMPA was selected based on previously published rodent studies (Tomita et al., 2020). Systolic blood pressure was measured on GD13 to GD19 by a noninvasive blood pressure analysis system, and all tests were repeated at least three times. On GD11 and GD19, mice were placed into metabolic cages to collect urine for 24 h for the measurement of the urinary albumin/creatinine ratio (ACR). On GD19, three mice from each group were sacrificed, and the tissues were harvested for further study. The remaining mice were allowed to spontaneously labor, and their offspring were observed up to 10 weeks to detect the survival rate and number. We recorded the body weight of offspring weekly and conducted whole-mount alcian blue and alizarin red staining to detect possible skeletal defects at 1 week of age in each of the six groups. The offspring were sacrificed at the age of 10 weeks at which time the tissues were harvested. In order to find out the susceptibility of preeclampsia mice to postpartum inciting factors, maternal mice in the control group, PE group, PE+losartan group and PE+EMPA group were injected with ADR (10 mg/kg) by tail vein at 12 weeks postpartum. ADR was used as an inciting factor here. The mice were sacrificed at 4 weeks after ADR injection (Figure 1A). Anesthesia was performed by inhalation of ether.

### Systolic Blood Pressure

SBP was measured with a computerized tail-cuff system in conscious mice (BP-2000 Blood Pressure Analysis System, Visitech System).

### Biochemical Parameters

Blood glucose was measured from the tail vein with a glucometer (Roche, Germany). Blood samples were centrifuged at 3,000 rpm for 15 min, and the supernatants were measured for BUN using a urea assay kit (R02904, Rayto Life and Analytical Sciences Co., Ltd., Shenzhen, China). Mouse urine was collected as described above. The urine samples were centrifuged at 3,000 rpm for 10 min, and the supernatants were measured for the ACR and glucose by an automatic biochemistry analyzer (HITACHI Roche Cobas 8,000, Japan). Urine levels of sodium were assessed using a sodium assay kit (C002–1-1, Nanjing Jiancheng Bioengineering Institute, Nanjing, China).

### Renal Histology

Kidneys were removed and fixed with 4% paraformaldehyde for 24 h at 4°C. 3-μm sections were cut from paraffin-embedded kidney tissues. Sections were stained with periodic acid-Schiff (PAS) and hematoxylin-eosin (HE) for histological analysis.

### Transmission Electron Microscopy

Mouse 1-mm^3^ renal cortex was fixed in 2.5% glutaraldehyde and postfixed with 1% osmium tetroxide (OsO4) in PBS for 1 h and dehydrated through a graded series of ethanol (30, 50, 70, 80, 95, 100, 100%) for 20 min at each step, followed by two changes of absolute acetone for 15 min. Tissue was placed into 1:1 mixture of acetone-EMBed 812 for 2–4 h, then into 1:2 mixture of acetone-EMBed 812 overnight at 37°C, followed by pure EMBed 812 for 5–8 h at 37°C. The embedding models were moved into a 65°C oven to polymerize for more than 48 h and cut to 60–880 nm thickness on an ultramicrotome. Finally, they were stained with 2.6% lead citrate for 8 min and examined with a transmission electron microscope (HITACHI, HT-7800, Japan).

### Immunofluorescence of Cells and Renal Tissues

Differentiated podocytes grown on coverslips were washed two times with ice-cold PBS and fixed in 4% formaldehyde solution for 15 min. After three washes with PBS, the cells were permeabilized with 0.1% Triton X-100 for 15 min followed by three washes with PBS. The podocytes were blocked in 5% BSA for 1 h. The primary antibodies were diluted in 1% BSA solution; the ratios of dilution were in accordance with the manufacturer’s instructions. Podocytes were incubated with primary antibodies overnight at 4°C. The following day, the cells were washed 3 times with PBS, incubated with fluorescently labeled secondary antibodies for 1 hour at room temperature, washed with PBS three times and labeled with DAPI. The cells were then mounted on microscope slides.

Mouse 3-μm paraffin-embedded kidney sections were deparaffinized and rehydrated in xylene with several exchanges in ethanol gradients. Antigen retrieval was performed with 10 mM Tris and 1 mM EDTA buffer. Endogenous peroxidase was blocked with 3% hydrogen peroxide. The tissues were permeabilized with 0.3% Triton X-100. Nonspecific binding was blocked with 5% goat serum in PBS. The primary antibodies were prepared in 1% BSA in PBS and incubated at 4°C overnight. The tissues were then incubated with fluorescently labeled secondary antibodies for 1 hour at room temperature and labeled with DAPI away from light. Fluorescence images were obtained using a Leica microscope (Leica DFC550, Germany).

### Whole-Mount Alcian Blue and Alizarin Red Staining

The offspring were sacrificed at 1 week of age, fixed in paraformaldehyde overnight and then in 95% ethanol for more than 5 days in whole mounts, and then treated with acetone for 2–7 days. Tissues were stained with alcian blue-alizarin red solution (0.3% Alcian blue: 0.1% Alizarin red: glacial acetic acid: 70% ethanol in 1:1:1:17) for 2–3 days, followed by incubation in 1% KOH until the soft tissue disintegrated (more than 2 days, with daily monitoring). The tissues were placed in 20% glycerol under transparent wrap for 1 week until the skeletons and muscles were translucent. The mice were transferred to 50% glycerol to take pictures. Whole body skeletons were captured using a Canon camera (EOS70D, Japan).

### Cell Culture and Treatments

A conditionally immortalized human podocyte cell line was donated by Professor Zhihong Liu (National Clinical Research Center for Kidney disease, Jinling Hospital, Medical School of Nanjing University, Nanjing, Jiangsu China). The podocytes were transfected with the temperature-sensitive SV40-T gene, which enables these cells to proliferate at 33°C, and differentiate at 37°C (Saleem et al., 2002). Podocytes were cultured at 33°C in RPMI-1640 medium (Sigma-Aldrich, United States) supplemented with 10% fetal bovine serum (FBS; Gibco, United States), 100 units/mL penicillin and 100 mg/ml streptomycin (Gibco, United States) and a mixture containing insulin, transferrin and selenium solution (ITS; Invitrogen, United States) for proliferation and then seeded into a collagen-coated 6-well plate and cultured at 37°C in a humidified atmosphere for 10–14 days for differentiation. The medium was exchanged every two or 3 days. To explore the effect of EMPA on AT1-AA-induced podocyte defects, the cells were seeded into a collagen-coated 6-well plate for differentiation and divided into four groups, namely the CTRL group (treated with nIgG), AT1-AA group (treated with 10 μg/ml AT1-AA for 48 h), AT1-AA+losartan group (treated with 1 μM losartan for 30 min, followed by incubation with 10 μg/ml AT1-AA for 48 h) and AT1-AA+EMPA group (treated with 1 μM EMPA for 30 min, followed by incubation with 10 μg/ml AT1-AA for 48 h).

### Cell Counting Kit-8

A total of 100 μL of cell suspension was seeded into 96-well plates in triplicate for the CCK-8 assay. 10 μL of CCK-8 solution (Dojindo, Japan) was added to each well. The plates were incubated in an incubator for 2 h; the absorbance at 450 nm was determined by a microplate reader (Tecan, infinite M200PRO, Switzerland).

### Measurement of Intracellular Reactive Oxygen Species Formation by Flow Cytometry in Human Podocytes

Human podocytes were seeded into a collagen-coated 6-well plate at a density of 1 × 10^5^ cells/well and then transferred to 37°C for 10–14 days for differentiation. Mature podocytes were starved overnight, divided into four groups and treated for 48 h as mentioned above. At the end of the treatments, intracellular ROS was determined using the 2′, 7′- dichlorodihydrofluorescein diacetate (H2DCFDA) method. Briefly, cells were washed with PBS and incubated in the dark at 37°C for 30 min in 1 ml serum-free media containing 10 µM DCFH-DA. After incubation, the fluorescence of 2, 7-dichlorofluorescein (DCF) was measured using an Amnis Flowsight multidimensional panoramic flow cytometer (Luminex, United States). The amount of ROS per cell was obtained as the mean fluorescence intensity (MFI) value. Samples were analyzed using FlowJo software (v10.6.2, United States).

### Western Blot Analysis

Mouse renal cortex total protein and cultured podocyte protein were extracted by using a RIPA: PMSF (100:1) mixture. After centrifugation, a BCA Kit (Beyotime P0009, Shanghai, China) was used to measure the protein concentration. The proteins were then denatured by heating to 100°C for 10 min, and equal amounts of 40 μg total protein were loaded and separated by SDS–PAGE. Proteins were transferred onto 0.22 μm PVDF membranes, blocked in 5% skim milk for 1 h and then incubated with primary antibodies overnight at 4°C. The following day, membranes were washed with TBST and then incubated with secondary antibodies for 2 h at room temperature. Finally, membranes were washed, and signals were recorded by using enhanced chemiluminescence (ECL).

### Quantitative Real Time Reverse Transcription–Polymerase Chain Reaction Analysis

Mouse renal cortex and cultured podocyte RNA was extracted by using Trizol Reagents (Invitrogen, United States). The PrimeScript™ RT Master Mix kit was used to generate cDNA. The cDNA was amplified by PCR using a TB Green Premix Ex Taq™ kit and the respective primers. The primer sequences were as follows: human *SGLT2* forward 5′-GGG​TTA​CGC​CTT​CCA​CGA​G-3′ and reverse 5′-AGA​TGT​TTC​CCA​CGG​CTG​G-3’; human *β-actin* forward 5′-CCTCGCCTTTGCCGA TCC-3′ and reverse 5′-CGC​GGC​GAT​ATC​ATC​ATC​C-3’; mouse *Sglt2* forward 5′-GCT​GCC​TAT​TTC​CTG​CTG​GT- 3′ and reverse 5′-GAA​CAG​AGA​GGC​TCC​AAC​CG-3’; and mouse *β-actin* forward 5′-CGC​AGC​CAC​TGT​CGA​GTC-3′ and reverse 5′-TCA​TCC​ATG​GCG​AAC​TGG​TG-3’. The amplification program was as follows: initial denaturation at 95°C for 30 s, then 40 cycles of denaturation at 95°C for 10 s and annealing at 60°C for 30 s. Gene expression was analyzed by the 2^−ΔΔCt^ method.

### Statistics

Data are reported as the mean ± SEM. Statistical significance was determined by one-way ANOVA with a Least Significant Difference (LSD) post hoc test for comparison of multiple groups. Differences with *p* < 0.05 were considered statistically significant.

## Results

### Empagliflozin’s Effects on Systemic and Laboratory Parameters in AT1-AA-Induced PE Mice

Maternal hypertension and proteinuria are the key clinical features of PE. To explore whether EMPA ameliorated preeclamptic manifestations in mice, mice were treated with vehicle or EMPA for 1 week after singly injecting AT1-AA into the tail vein (Figure 1A). The angiotensin Ⅱ receptor blocker (ARB) inhibitor losartan was used as a standard antihypertensive therapy. Mice that underwent AT1-AA infusion showed typical preeclamptic features, such as high systolic blood pressure (Figure 1B) and elevated urinary ACR (Figure 1C). Oral administration of EMPA and losartan significantly improved maternal outcomes in the PE model. Specifically, the systolic blood pressure and urinary ACR were significantly reduced in losartan- and EMPA-treated mice (Figures 1B,C). EMPA, a SGLT2 inhibitor, was originally developed as an antihyperglycemic agent and has been shown to have a weight loss effect in type 2 diabetes patients (Vasilakou et al., 2013). Here, we show that oral administration of EMPA caused transient weight loss and had no effect on normal blood glucose in PE mice (Figures 1D,E). Blood urea nitrogen (BUN) was higher in EMPA-treated mice than in other groups (Figure 1F). Treatment with EMPA induced glycosuria and natriuresis in PE mice (Figures 1G,H).

### Empagliflozin Limited Kidney and Podocyte Damage in Mice With AT1-AA-Induced Preeclampsia

**FIGURE 2:**
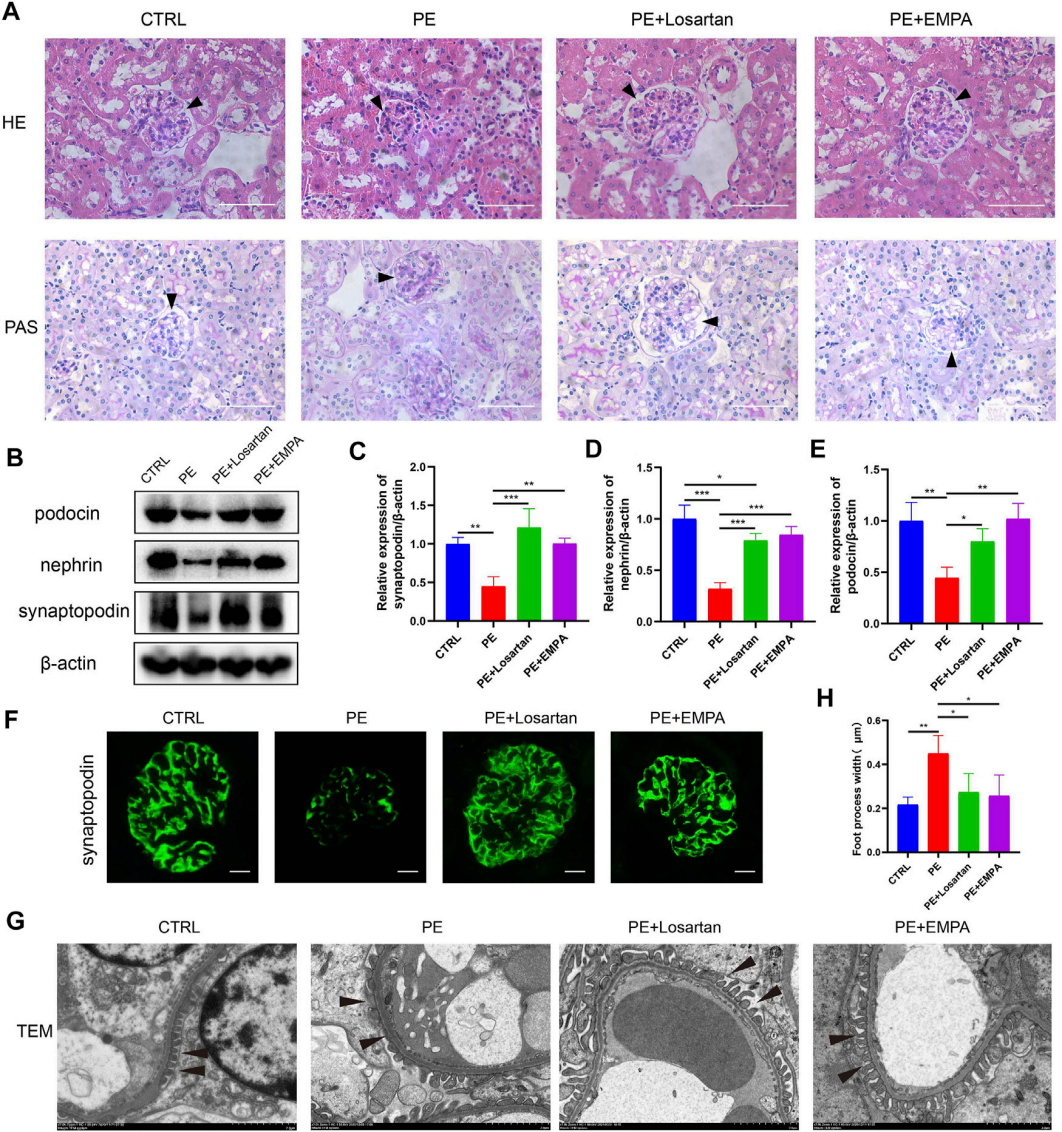
EMPA ameliorated renal injury and podocytopathy in AT1-AA-induced PE mice. **(A)** Representative hematoxylin-eosin (HE) and periodic acid-Schiff (PAS) images of kidneys from different groups (scale bar represents 50 μm), Bowman’s spaces are indicated by arrowheads. **(B)** Representative Western blots for synaptopodin, nephrin and podocin in the glomeruli of kidney sections. Semiquantitative analysis of Western blots for **(C)** synaptopodin (*n* = 3), **(D)** nephrin (*n* = 3) and **(E)** podocin (*n* = 3). **(F)** Representative immunofluorescence images of synaptopodin in kidneys of different groups. Scale bar: 10 μm. **(G)** Representative electron micrographs of glomeruli from control and PE mice treated with vehicle, EMPA or losartan. Podocyte foot processes are indicated by arrowheads. Scale bar: 2 μm. **(H)** Quantitative podocyte foot process width (*n* = 3). Data are expressed as the mean ± SEM and were analyzed by ANOVA with LSD post hoc test. *p < 0.05, **p < 0.01, ***p < 0.001.

AT1-AA-induced PE mice showed kidney histopathological alterations, such as narrowing of Bowman’s space and vascular congestion (Figure 2A). Oral administration of EMPA and losartan improved kidney histopathology in the PE model. Podocyte damage is another feature of PE. Nephrin and podocin are key podocyte proteins in the slit diaphragm. Synaptopodin is an actin-associated protein that regulates the actin cytoskeleton in podocytes, and defects in these proteins lead to proteinuria (Yanagida-Asanuma et al., 2007; Martin & Jones, 2018). As shown by Western blots, the expression of synaptopodin, nephrin and podocin in PE mouse renal cells was lower than that in normal pregnant mice (Figures 2B–E). Representative immunofluorescence images also showed decreased glomerular synaptopodin expression in PE mice (Figure 2F). Losartan and EMPA treatment ameliorated the defective expression of synaptopodin, nephrin and podocin (Figures 2B–F). Ultrastructural analysis showed intact foot processes of podocytes in control mouse kidneys and showed focal areas of damaged podocytes with foot process effacement in PE mice (Figures 2G,H). These ultrastructural changes were infrequent in mice treated with EMPA or losartan.

### Empagliflozin Ameliorated AT1-AA-Induced Podocyte Injury, Intracellular Reactive Oxygen Species Accumulation and Cytoskeletal Remodeling in Cultured Podocytes

**FIGURE 3:**
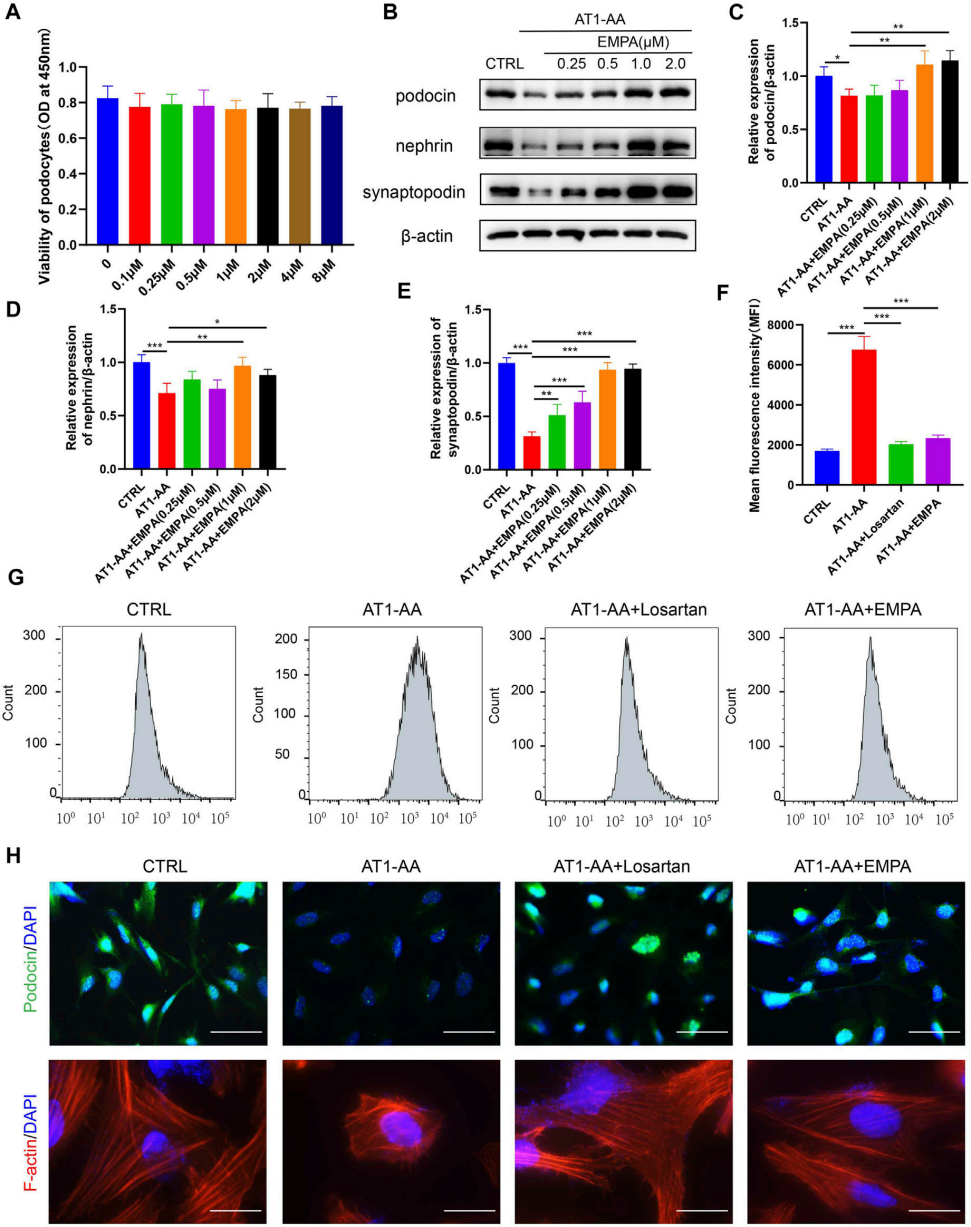
EMPA limited AT1-AA-induced podocyte damage *in vitro*. **(A)** CCK8 assays of the effect of EMPA on podocyte viability (*n* = 3). **(B)** Representative Western blots for synaptopodin, nephrin and podocin in cultured podocytes. Semiquantitative analysis of Western blots for **(C)** synaptopodin (*n* = 3), **(D)** nephrin (*n* = 3) and **(E)** podocin (*n* = 3). **(F)** The graph displays the mean fluorescence intensities (MFIs) obtained from flow cytometric analysis of intracellular ROS (*n* = 3). **(G)** Representative flow cytometry histogram for DCF in the four groups. **(H)** Representative images of immunofluorescence staining for podocin and F-actin. Scale bars: upper panel, 50 μm; lower panel, 20 μm. Data are expressed as the mean ± SEM and were analyzed by ANOVA with LSD post hoc test. *p < 0.05, **p < 0.01, ***p < 0.001.

The above results indicate that EMPA ameliorates preeclamptic symptoms in AT1-AA-induced PE mice. We further studied the effect of EMPA on cultured podocytes. We first evaluated possible detrimental effects of EMPA on podocytes. CCK-8 assays showed that 0.1–8 μM EMPA had no significant effect on podocyte viability (Figure 3A). Next, conditionally immortalized human podocytes were incubated with nIgG (CTRL), AT1-AA or AT1-AA plus EMPA for 48 h at EMPA concentrations of 0.25, 0.5, 1.0 or 2 μM. Western blotting and immunofluorescence revealed that AT1-AA decreased the expression of synaptopodin, nephrin and podocin (Figures 3B–E,H). EMPA limited the reduced expression of podocyte-specific markers in a concentration-dependent manner, reaching a plateau at a 1.0 μM concentration (Figures 3B–E); thus, this concentration was used for further experiments. We additionally studied the effects of EMPA on AT1-AA-induced intracellular ROS accumulation in podocytes; the level of ROS in podocytes was quantified by CM-H2DCFDA using flow cytometric analysis. The level of ROS in podocytes treated with AT1-AA for 48 h alone increased nearly 3-fold compared with that in control podocytes (Figures 3F,G). This increase in ROS was significantly suppressed by pretreatment (0.5 h) with EMPA or losartan (Figures 3F,G). These results demonstrate that EMPA has potent antioxidant effects. The function of podocytes is based largely on their complex architecture, in particular on the maintenance of highly ordered, parallel, contractile actin filament bundles in FPs (Cosentino et al., 2020). Interference with FP domains changes the actin cytoskeleton from parallel, contractile bundles to a dense network and results in FP effacement and proteinuria (Kerjaschki, 2001). Immunofluorescence assay of F-actin indicated that AT1-AA induced podocyte cytoskeletal remodeling (Figure 3H). EMPA and losartan treatment ameliorated the podocyte cytoskeletal remodeling induced by AT1-AA (Figure 3H).

### Podocyte Expression of SGLT2 Is Upregulated by AT1-AA Induction *In Vivo* and *In Vitro*

**FIGURE 4:**
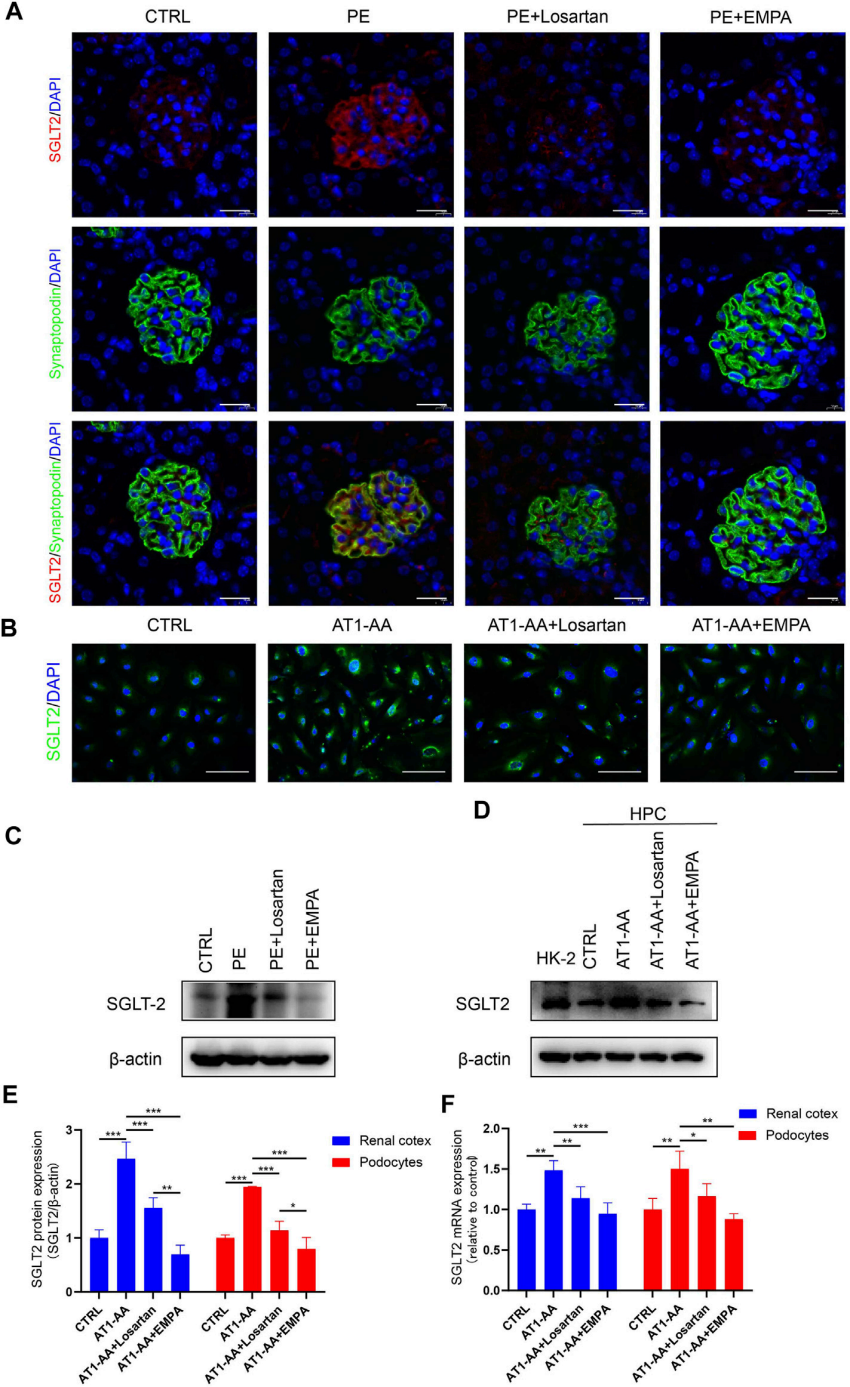
SGLT2 is expressed in podocytes and upregulated by AT1-AA induction *in vivo* and *in vitro*. **(A)** Representative fluorescence pictures of SGLT2 (red) and synaptopodin (green) in control and PE mouse glomeruli. Sections were counterstained for DNA (blue). Scale bar: 20 μm. **(B)** Representative images of immunofluorescence staining for SGLT2 (green) in podocytes. Scale bar: 50 μm. **(C)** Representative Western blots and **(E)** densitometric analysis of SGLT2 protein in renal cortex. **(D)** Representative Western blots and **(E)** densitometric analysis of SGLT2 protein in cultured human podocytes (*n* = 3). **(F)** Podocytes SGLT2 and mouse renal cortex Sglt2 mRNA expression was evaluated by real-time qPCR analysis (*n* = 3). Data are expressed as the mean ± SEM and were analyzed by ANOVA with LSD post hoc test. *p < 0.05, **p < 0.01, ***p < 0.001.

The fact that EMPA directly protects against AT1-AA-induced podocyte injury inspired us to explore the expression of SGLT2 in podocytes. A previous study showed that SGLT2 is expressed in mouse and human podocytes and is upregulated in podocytes from patients with CKD (Cassis et al., 2018). Immunofluorescence analysis of kidney sections from pregnant control mice showed weak glomerular staining of SGLT2 (Figure 4A). Western blots and PCR also showed low level expression of SGLT2 protein and *Sglt2* mRNA in renal cortices from control mice (Figures 4A,C,E,F). On the other hand, AT1-AA-induced PE mice showed a more intense signal in glomeruli; significantly, highly expressed SGLT2 was mainly localized to podocytes, as shown by co-staining of SGLT2 protein with the podocyte marker synaptopodin (Figure 4A). Losartan and EMPA treatment reduced the expression of SGLT2 (Figures 4A,C,E,F). Having established that AT1-AA upregulated podocyte expression of SGLT2 *in vivo*, we further investigated the expression of SGLT2 in cultured human podocytes. To verify the effectiveness of the SGLT2 antibody, human renal tubular epithelial cells (HK2) were used as a control. Incubation with AT1-AA upregulated podocyte SGLT2 protein expression and *SGLT2* mRNA expression. Both EMPA and losartan were able to lower SGLT2 expression to varying degrees (Figures 4B,D–F).

### Empagliflozin Activates AMPK/SIRT1 and Limits Oxidative Stress in AT1-AA-Induced Preeclampsia Mouse Kidneys

**FIGURE 5:**
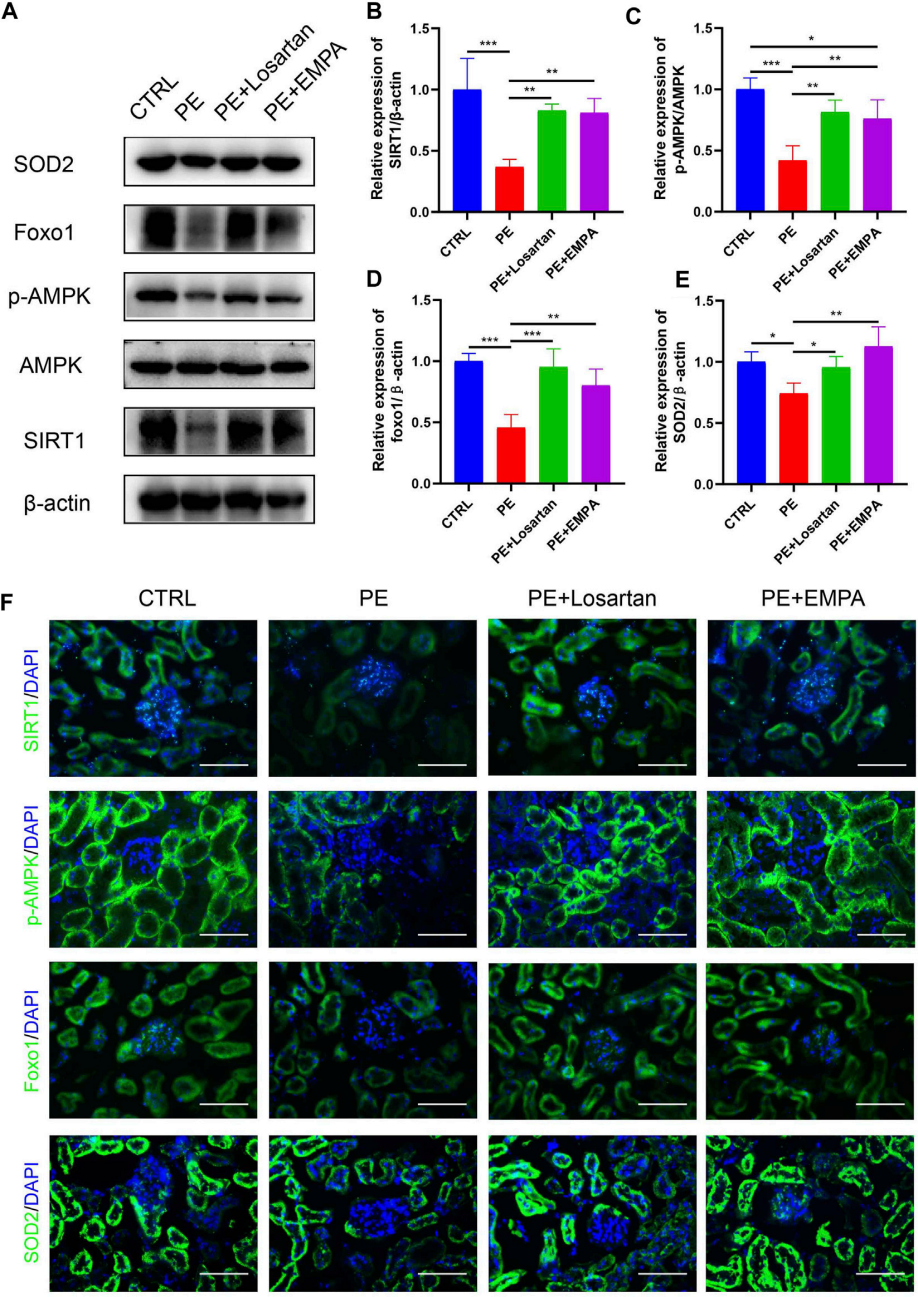
Effects of EMPA on the AMPK/SIRT1 pathway and oxidative stress in AT1-AA-induced PE mouse kidneys. **(A)** Protein levels of SIRT1, AMPK, p-AMPK, Foxo1 and SOD2 in the kidney determined by Western blot analysis. **(B)** Densitometric evaluation of Western blots for SIRT1 (*n* = 3), **(C)** p-AMPK (*n* = 3), **(D)** Foxo1 (*n* = 3) and **(E)** SOD2 (*n* = 3). **(F)** Representative immunofluorescence images of SIRT1, p-AMPK, Foxo1 and SOD2 in kidneys of different groups. Scale bar: 50 μm. Data are expressed as the mean ± SEM and were analyzed by ANOVA with LSD post hoc test. *p < 0.05, **p < 0.01, ***p < 0.001.

SGLT2 inhibitors have been shown to have reno-protective effects, regardless of the presence or absence of diabetes, in DAPA-CKD (Heerspink et al., 2020). However, the exact reno-protective mechanism remains obscure. From a metabolic perspective, SGLT2 inhibitors promote glucosuria and modestly contribute to glycemic control (Neuen et al., 2019). Glucosuria also enhances gluconeogenesis and ketogenesis, indicating that SGLT2 inhibitors induce a fasting-like metabolic and transcriptional paradigm that mimics nutrient and oxygen deprivation (Osataphan et al., 2019). Specific sensors, such as SIRT1 and AMPK, are activated in response to nutrient deprivation (Packer, 2020a). SIRT1 serves as a redox rheostat and represents a critical molecular response to caloric restriction, such as FOXO1 (Horio et al., 2011). FOXO1 is important for cell survival by transactivating ROS-detoxifying enzyme superoxide dismutase 2 (SOD2/MnSOD) (Huang & Tindall, 2007). A previous study showed that SIRT1 was downregulated in the plasma of PE patients and in HUVECs incubated with plasma from PE patients (Viana-Mattioli et al., 2020), indicating a possible underlying pathophysiologic mechanism of SIRT1 in PE. Therefore, we hypothesized that the SGLT2 inhibitor EMPA would activate AMPK/SIRT1 and exert antioxidant effects in AT1-AA-induced PE mice. Western blot and immunofluorescence images indicated that AT1-AA downregulated p-AMPK, SIRT1, FOXO1 and SOD2 in mouse kidneys. On the other hand, EMPA and losartan promoted the phosphorylation of AMPK and upregulated SIRT1, FOXO1 and SOD2 in mouse kidneys (Figures 5A–F).

### Empagliflozin Activates AMPK/SIRT1 and Limits Oxidative Stress in Cultured Human Podocytes

**FIGURE 6:**
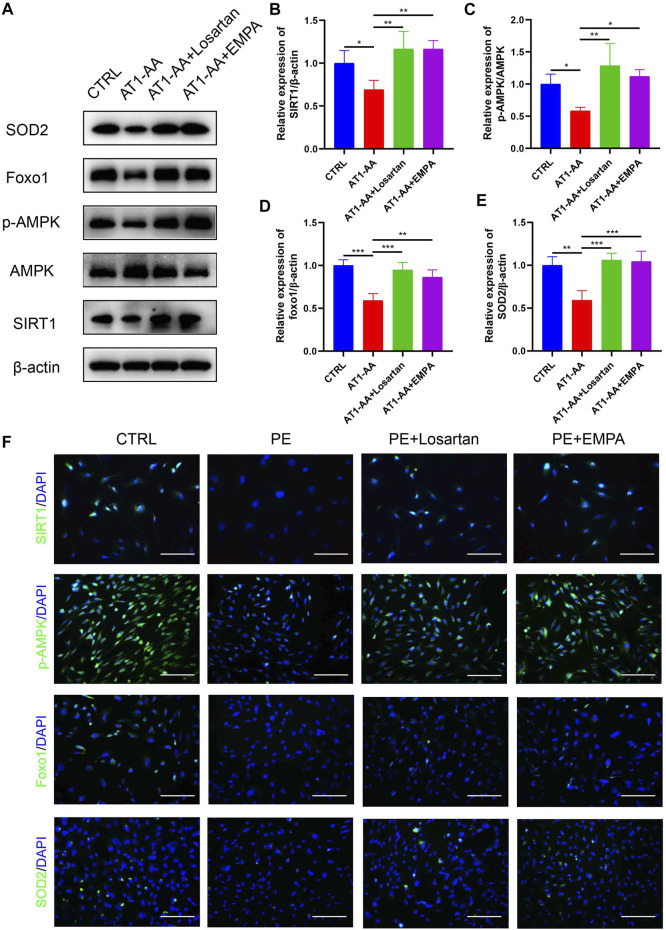
Effects of EMPA on AMPK/SIRT1 and oxidative stress in cultured human podocytes. **(A)** Protein levels of SIRT1, AMPK, p-AMPK, Foxo1 and SOD2 in podocytes determined by Western blot analysis. **(B)** Densitometric evaluation of Western blots for SIRT1 (*n* = 3), **(C)** p-AMPK (*n* = 3), **(D)** Foxo1 (*n* = 3) and **(E)** SOD2 (*n* = 3). **(F)** Representative immunofluorescence images of SIRT1, p-AMPK, Foxo1 and SOD2 in podocytes. Scale bar: 100 μm. Data are expressed as the mean ± SEM and were analyzed by ANOVA with LSD post hoc test. *p < 0.05, **p < 0.01, ***p < 0.001.

We further explored the effect of EMPA on AMPK/SIRT1 and oxidative stress *in vitro*. In line with the *in vivo* data, AT1-AA downregulated the expression of p-AMPK, SIRT1, FOXO1 and SOD2 in cultured podocytes, as shown by Western blots and immunofluorescence. EMPA and losartan promoted the phosphorylation of AMPK and upregulated SIRT1, FOXO1 and SOD2 expression to differing degrees. (Figures 6A–F).

### The Effect of Empagliflozin on Fetal Outcomes in AT1-AA-Induced PE Mice

**FIGURE 7:**
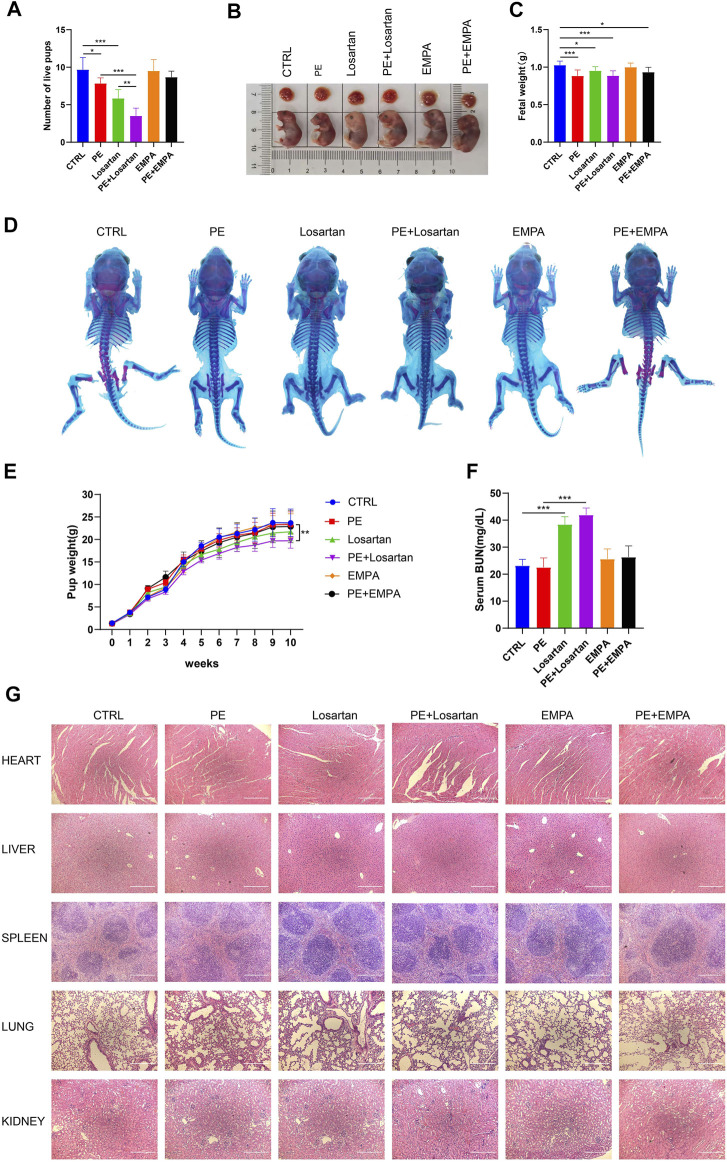
Effects of EMPA on fetal outcomes in PE mice. **(A)** Histogram showing the number of live pups in each group (*n* = 6). **(B)** Representative images showing the gross morphology of placentae (top) and embryos (bottom) in each group. **(C)** Histogram showing the fetal weight in each group (*n* = 6). **(D)** Whole-mount alcian blue and alizarin red staining of 1-week-old offspring in each group. **(E)** Pup weights recorded from 0 to 10 weeks of each group (*n* = 8). **(F)** Serum BUN levels of each group of offspring at 10 weeks of age (*n* = 6). **(G)** H&E staining of hearts, livers, spleens, lungs and kidneys of offspring mice in each group. Scale bar: 200 μm. The results are expressed as the mean ± SEM. *p < 0.05, **p < 0.01, ***p < 0.001.

Due to concerns for maternal, fetal and infant safety, medications during pregnancy are mostly restricted (Roset Bahmanyar et al., 2021). Therefore, we undertook to observe the effect of EMPA and losartan on fetal outcomes. Offspring were from CTRL mice, AT1-AA-induced PE mice, losartan-treated pregnant mice, losartan-treated PE mice, EMPA-treated pregnant mice and EMPA-treated PE mice. The number of live pups was reduced in AT1-AA-induced PE mice (Figure 7A); EMPA treatment had no negative effects on the survival of offspring in normal pregnant mice or in PE mice (Figure 7A); and losartan treatment decreased the number of live pups in normal pregnant mice and in PE mice (Figure 7A). Fetal weight in the PE group was lower than that in the control group (Figures 7B,C). Losartan treatment lowered fetal weight in normal pregnant mice (Figures 7B,C). No difference in whole skeleton (Figure 7D) or H&E staining of fetal organs (Figure 7G) was observed among the six groups. The birth weight of pups in the PE group was lower than that in the control group (Figure 7E). As they grew, pup weight was not significantly different between the control and PE groups at 1 week of age (Figure 7E). The birth weight of pups in the losartan-treated PE group was lower than that in the PE group; this finding persisted into adulthood (Figure 7E). EMPA had no significant effect on pup weights in normal pregnant mice or in PE mice (Figure 7E). There were no significant differences in serum BUN levels of offspring in the control or PE groups (Figure 7F). Losartan treatment increased serum BUN levels of offspring in control and PE mice; EMPA had no obvious effects on serum BUN levels of offspring (Figure 7F).

### Empagliflozin Reduced Postpartum Susceptibility to Adriamycin in AT1-AA-Injected Mice

**FIGURE 8:**
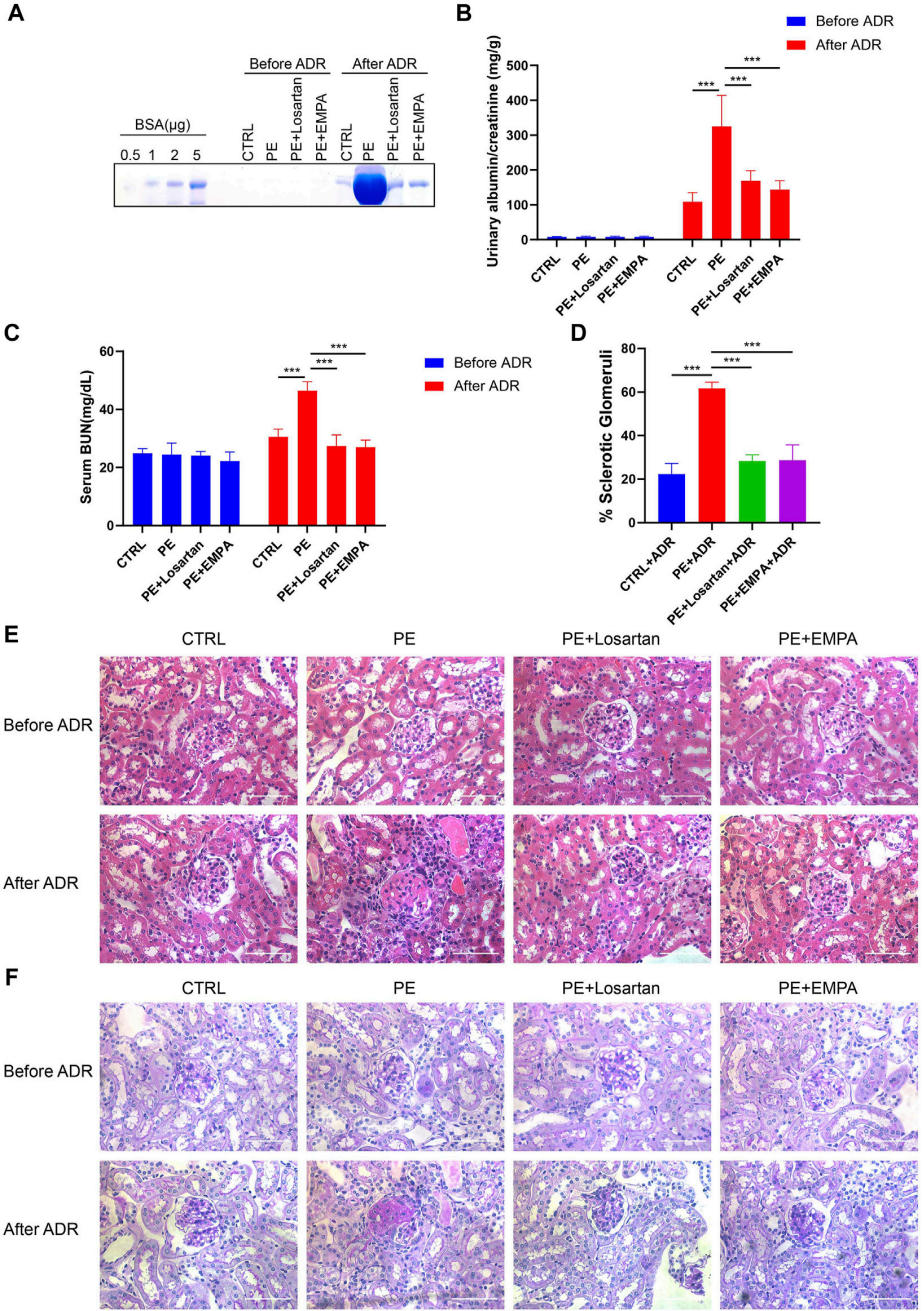
EMPA treatment during pregnancy limited the postpartum renal injury response to ADR in AT1-AA-injected maternal mice. **(A)** SDS–PAGE Coomassie blue staining of bovine serum albumin (BSA) standards and urine from postpartum mice before and after ADR injection, demonstrating proteinuria in these mice; 16 μL of each urine sample was loaded. **(B)** Quantification of urinary ACRs of postpartum mice before (*n* = 9) and after ADR (*n* = 6) injection. **(C)** Serum BUN levels of PE mice before (*n* = 3) and after ADR (*n* = 6) injection. **(D)** Quantification of the percentage of sclerotic glomeruli in PE mice 4 weeks after ADR injection (*n* = 3). **(E)** Representative H&E staining and **(F)** PAS staining of kidney sections in postpartum mice before and after ADR injection (scale bar represents 50 μm). Data are expressed as the mean ± SEM and were analyzed by ANOVA with LSD post hoc test. *p < 0.05, **p < 0.01, ***p < 0.001.

It has been noted that women with PE are at a 5- to 12-fold increased risk of developing end-stage renal disease (ESRD) (Vikse et al., 2008). We wondered whether temporary treatment with EMPA during pregnancy would reduce postpartum susceptibility to inciting factors in PE mice. Adriamycin nephropathy (AN) is a rodent model of chronic kidney disease that is characterized by podocyte injury followed by glomerulosclerosis, and a number of factors alter both the risk and severity of renal injury induced by ADR (Lee & Harris, 2011). Therefore, AT1-AA-injected mice that were treated with losartan or EMPA during pregnancy were subjected to ADR stimulation at 12 weeks postpartum and were studied for 4 weeks after ADR injection. The urinary albumin excretion and renal histopathology of AT1-AA-injected mice returned to normal at 12 weeks postpartum, as shown by SDS-PAGE Coomassie blue staining of urine from control and PE mice (Figure 8A), urinary ACR (Figure 8B), serum BUN levels (Figure 8C) and renal cortex staining of HE and PAS (Figures 8E,F). When we challenged PE mice with ADR, PE mice showed elevated urinary albumin and serum BUN levels compared to control mice (Figures 8A–C), and renal histopathology showed a higher percentage of glomerulosclerosis than in control mice (Figures 8D–F). EMPA and losartan treatment during pregnancy limited the elevated urinary albumin and serum BUN levels and glomerulosclerosis in postpartum PE mice (Figures 8A–F).

**FIGURE 9:**
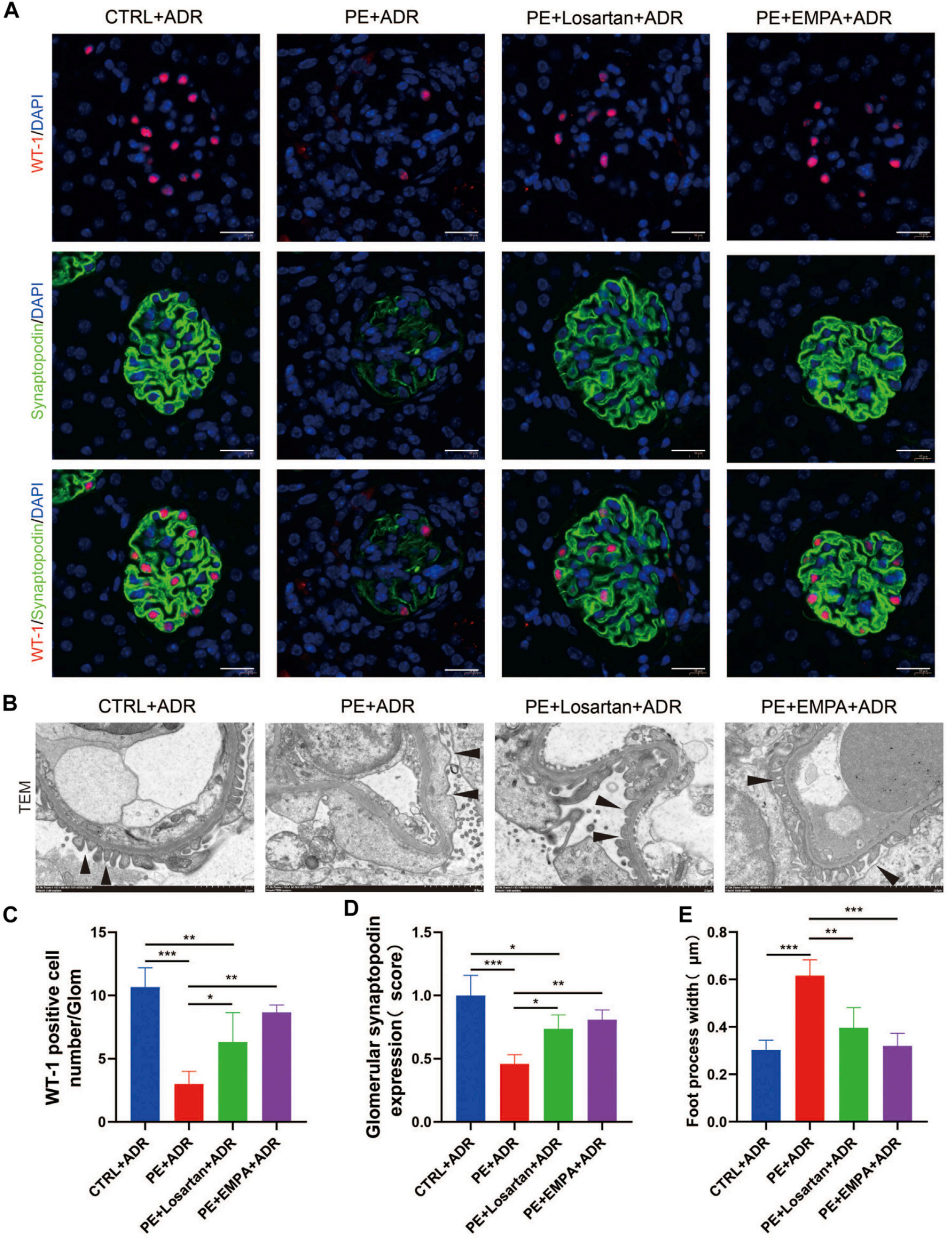
EMPA treatment during pregnancy limited postpartum glomerular podocyte damage in response to ADR in AT1-AA-injected maternal mice. **(A)** Representative pictures of WT1-positive podocytes (red) and synaptopodin (green) in mouse glomeruli after ADR injection. Sections were counterstained for DNA (blue). Scale bar: 20 μm. **(B)** Representative transmission electron microscopy (TEM) micrographs of glomeruli from ADR-stimulated postpartum mouse kidneys reveal podocyte ultrastructure changes; podocyte foot processes are indicated by arrowheads. Scale bar: 2 μm. **(C)** Quantification of WT-1-positive cells/glomerulus (*n* = 3) and **(D)** synaptopodin expression (*n* = 3) in mouse glomeruli after ADR injection. **(E)** Quantitative podocyte foot process width (*n* = 3). Data are expressed as the mean ± SEM and were analyzed by ANOVA with LSD post hoc test. *p < 0.05, **p < 0.01, ***p < 0.001.

We further explored the protective effect of EMPA treatment during pregnancy on postpartum podocyte damage induced by ADR in AT1-AA-injected maternal mice. WT-1 is specifically expressed in podocyte nuclei in mature kidneys and plays a major role in the maintenance of podocyte function (Dong et al., 2015); thus, WT-1 was used as a marker for podocyte nuclei. We carried out co-staining of synaptopodin and WT-1 in PE mouse kidneys and found that postpartum mice with PE showed lower expression of synaptopodin (Figures 9A,D), a decreased number of WT-1-positive cells per glomerulus (Figures 9A,C), more severe podocyte FP broadening and effacement and glomerular basement membrane thickening (Figures 9B,E). The PE mice treated with EMPA and losartan during pregnancy showed reduced loss of podocyte synaptopodin and of WT-1-positive cells and fewer podocyte ultrastructure mutations (Figures 9A–E). The protective effect of EMPA on postpartum podocyte damage was better than that of losartan, although no significant difference was found.

## Discussion

Large randomized clinical controlled trials have demonstrated both renal and cardiovascular protection in patients with or without type 2 diabetes. However, translational studies of SGLT2 inhibition in nondiabetic kidney disease are still lacking. The present study investigated the effects of EMPA treatment on AT1-AA-induced PE. The results showed that EMPA treatment is able to reduce high SBP and proteinuria, and improve kidney histopathology, thereby, improving PE without affecting fetal outcomes. Furthermore, we found that EMPA treatment ameliorated podocyte injury induced by AT1-AA both *in vivo* and *in vitro,* and reduced intracellular ROS accumulation in podocytes. Furthermore, EMPA treatment reduced the expression of SGLT2, improved oxidative stress and activated the AMPK/SIRT1 signaling pathway. In addition, EMPA treatment during pregnancy limited the elevation of urinary albumin and serum BUN levels, glomerulosclerosis and podocyte damage in response to ADR in postpartum PE mice. Last but not least, EMPA did not affect fetal outcomes.

As mammals differ in the placentation process, it is difficult to find a single reliable animal model that recapitulates all aspects of PE (Martinez-Fierro et al., 2018). To achieve a better understanding of the pathophysiology of PE, several models have been established, one of which is induced by AT1-AA and has been demonstrated to stimulate angiotensin II type 1 receptors and exhibiting agonistic effects similar to those of angiotensin II (Wallukat et al., 1999). AT1-AAs are upregulated in the serum of preeclamptic women, and antibody titers to AT1-AA correlate with disease severity (Dechend et al., 2005; Siddiqui et al., 2010). Studies have shown that injection of AT1-AA induces key features of PE, including hypertension, proteinuria, glomerular endotheliosis, reduced fetal weights, placental abnormalities and increased circulating sFlt-1 and sEng (Zhou et al., 2008; Zhou et al., 2010; Parrish et al., 2011). AT1-AAs also increase the production of ROS (Zhou et al., 2010) and tumor necrosis factor alpha (TNF-α) (Irani et al., 2010) and increase renal vascular resistance (RVR) (Cunningham et al., 2016) in AT1-AA-infused pregnant rodents, all of which contribute to PE. In the present study, we injected AT1-AA derived from guinea pigs into pregnant mice and induced typical preeclamptic features, such as hypertension, proteinuria, renal pathological changes and reduced fetal weights. Podocyte and SD damage has been proposed as additional features of PE, and urinary nephrin has even been recommended as a marker for the development of PE due to its high sensitivity and specificity (Kerley and McCarthy, 2018). Our previous work found that AT1-AA isolated from preeclamptic sera induced podocyte damage *in vitro* (Yu et al., 2018). In the current study, AT1-AA induced podocyte injury *in vivo* and *in vitro*. These results indicate that our AT1-AA-induced PE mouse model is reliable and properly replicates the pathological changes of PE in human beings.

It has been shown that AT1-AA-induced preeclamptic features are preventable by co-injection with losartan, an AT1 receptor antagonist, or by 7 aa, an antibody neutralizing, seven amino acid epitope peptide (Zhou et al., 2008). Angiotensin-converting enzyme inhibitors and angiotensin receptor antagonists are banned in pregnant women due to neonatal and long-term complications (Bullo et al., 2012). The safety of modified peptide sequences that bind AT1-AAs is under study. In the current study, losartan improved all the clinical manifestations of PE; however, we found a low number of live pups, low fetal and adult weights and elevated serum BUN levels in the offspring of PE mice that were treated with losartan during pregnancy, confirming that losartan is not a suitable choice for pregnant women.

The current treatments for obstetrical complications are very limited due to the challenges of drug development in pregnancy (Roset Bahmanyar et al., 2021). Here, we investigated the effects of EMPA, an SGLT2 inhibitor, on PE. SGLT2 inhibitors were developed as glucose-lowering agents and have been shown to have renal and cardiovascular protective effects (Perkovic et al., 2019; Heerspink et al., 2020; Packer et al., 2020). Beyond glycemic control, SGLT2 inhibitors have been shown to have pleiotropic effects on reno-protective mechanisms. SGLT2 inhibitors regulate glomerular hemodynamics through their effects on: 1) natriuresis and tubulo-glomerular feedback (Heerspink et al., 2018); 2) increasing glucose excretion, alleviating metabolic burden in nephrons (Mima, 2018); 3) lowering blood pressure due to diuresis, natriuresis and sympatholytic effects (Baker et al., 2014; Wan et al., 2018); 4) and lowering body weight due to glucosuria and ketogenesis-induced caloric loss (Neuen et al., 2019). In the current research, we observed the decreases in blood pressure, body weight and glucosuria, and natriuresis effects due to EMPA in PE mice. In particular, EMPA-induced loss of body weight was only temporary and did not affect maternal weight thereafter or impact fetal outcomes. Although EMPA has an antihyperglycemic effect in diabetic patients, we did not observe blood sugar reduction by EMPA in normal glucose mice. We also found a slight elevation in serum BUN in EMPA-treated maternal mice, which may be due to the short-term loss of body fluids and natriuresis induced by EMPA. The fact that the serum BUN levels of maternal mice returned to normal at 12 weeks postpartum reinforced our theory, indicating that the SGLT2i-induced diuretic effect can be adequately mitigated through an efficacious physiological response. EMPA also showed reno-protective effects in PE mice during pregnancy and postpartum. In particular, we found that EMPA reduced proteinuria and podocyte dysfunction in PE mice. SGLT2 inhibitors have been shown to attenuate podocyte damage and proteinuria in diabetic db/db mice (Tomita et al., 2020) and in nondiabetic chronic kidney disease mice (Cassis et al., 2018). They also showed that SGLT2 was expressed in podocytes and upregulated after BSA injections (Cassis et al., 2018), indicating that SGLT2 inhibitors may directly target podocytes and exert protective effects. Our current study indicated that SGLT2 is expressed in podocytes and upregulated in AT1-AA-infused pregnant mice and cultured podocytes. These results indicate that podocyte SGLT2 may participate in the pathogenesis of PE.

The mechanism of podocyte damage in PE is connected with oxidative stress, and the expression of the antioxidant CuZn-SOD was lower in podocytes shed from preeclamptic patients (Wang et al., 2018). Normal pregnancy is characterized as a pro-oxidant period, while PE exacerbates this process. The mechanisms of this process are connected with placental ischemia reperfusion and an increase in TNF-α in maternal circulation (Tenório et al., 2019). Oxidative stress in PE has been associated with the reduction of SIRT1 levels and activity (Salminen et al., 2013). SIRT1 has been demonstrated to be downregulated in the plasma of PE patients and in HUVECs incubated with plasma from PE patients (Viana-Mattioli et al., 2020). In PE patients, maternal serum p-AMPK is positively correlated with the severity of PE and BP (Koroglu et al., 2019). Previous studies have shown that metformin is able to activate SIRT1 and AMPK and decrease sFlt-1 expression and secretion as a PE treatment (Hastie et al., 2019), indicating that SIRT1 and AMPK are potential therapeutic targets of PE. Numerous studies have proven that SIRT1 can deacetylate FOXO factors, such as FOXO1 and FOXO3a, and subsequently stimulate the expression of the antioxidant MnSOD, thereby potentiating SIRT1 expression via an auto-feedback loop (Brunet et al., 2004; Xiong et al., 2011). SGLT2 inhibitors are able to induce a fasting-like transcriptional paradigm, which is characterized by loss of calories in the urine, shrinkage of adipose tissue depots and promotion of gluconeogenesis and ketogenesis (Packer, 2020b); thus, nutrient deprivation signaling, such as by SIRT1 and AMPK, is stimulated (Packer, 2020a). SGLT2 inhibitors have also been shown to prevent oxidative stress in diabetic rats (Osorio et al., 2012). The mechanism by which SGLT2 inhibitors alleviate oxidative stress may be related to their promotion of increased ketone production (Mima, 2021). Therefore, we speculated that in PE the kidneys and podocytes would exhibit lower expression of SIRT1 and AMPK as well as elevated oxidative stress, and SGLT2 inhibitors might be able to reverse these effects. In the current study, we found elevated ROS production in AT1-AA-induced podocytes. We also found lower expression of the antioxidant SOD2 in AT1-AA-infused pregnant mouse kidneys and cultured podocytes and downregulation of the SIRT1/AMPK pathway and the downstream factor FOXO1. These adverse effects were reversed by EMPA treatment. This might explain the partial mechanisms of the renal protective effects of SGLT2 inhibitors on PE.

To our knowledge, this is the first study investigating whether supplementation with SGLT2 inhibitors during pregnancy has adverse effects on fetal outcomes. In our study, treatment with EMPA at a dosage of 30 mg/kg/d from gestation Day 13 to gestation Day 19 did not cause significant fetal abnormalities or growth defects. This will provide some background for the use of SGLT2 inhibitors in a larger population. Even so, the safety of other SGLT2 inhibitors throughout the entirety of pregnancy needs further investigation.

There are still some shortcomings in our study. In the current research, although we have proven that EMPA administration during pregnancy can reduce the susceptibility of AT1-AA-injected maternal mice to ADR after delivery, the mechanisms of increased renal susceptibility to ADR after PE remain unclear, and further investigation is needed so we can take timely and effective measures for intervention. PE is a multisystem disease, and we mainly focused on the kidneys. Studies of other systems, including the placenta, are lacking in our study, which is also a deficiency of this study.

## Conclusion

In summary, we have shown that EMPA activates the AMPK/SIRT1 signaling pathway, suppresses oxidative stress and ameliorates podocyte injury, thus improving the short- and long-term prognoses of PE without affecting offspring. These findings suggest that EMPA could be a potential pharmacological agent for PE therapy.

## Data Availability

The original contributions presented in the study are included in the article/Supplementary Material, further inquiries can be directed to the corresponding author.
