# Supplementary material for: Empagliflozin Ameliorates Preeclampsia and Reduces Postpartum Susceptibility to Adriamycin in a Mouse Model Induced by Angiotensin Receptor Agonistic Autoantibodies
Source: Front Pharmacol. 2022 Mar 23;13:826792. doi: 10.3389/fphar.2022.826792 (PMC8984158; doi:10.3389/fphar.2022.826792)
Supplement: Supplementary file 1 [file DataSheet1.PDF]

Figure2B

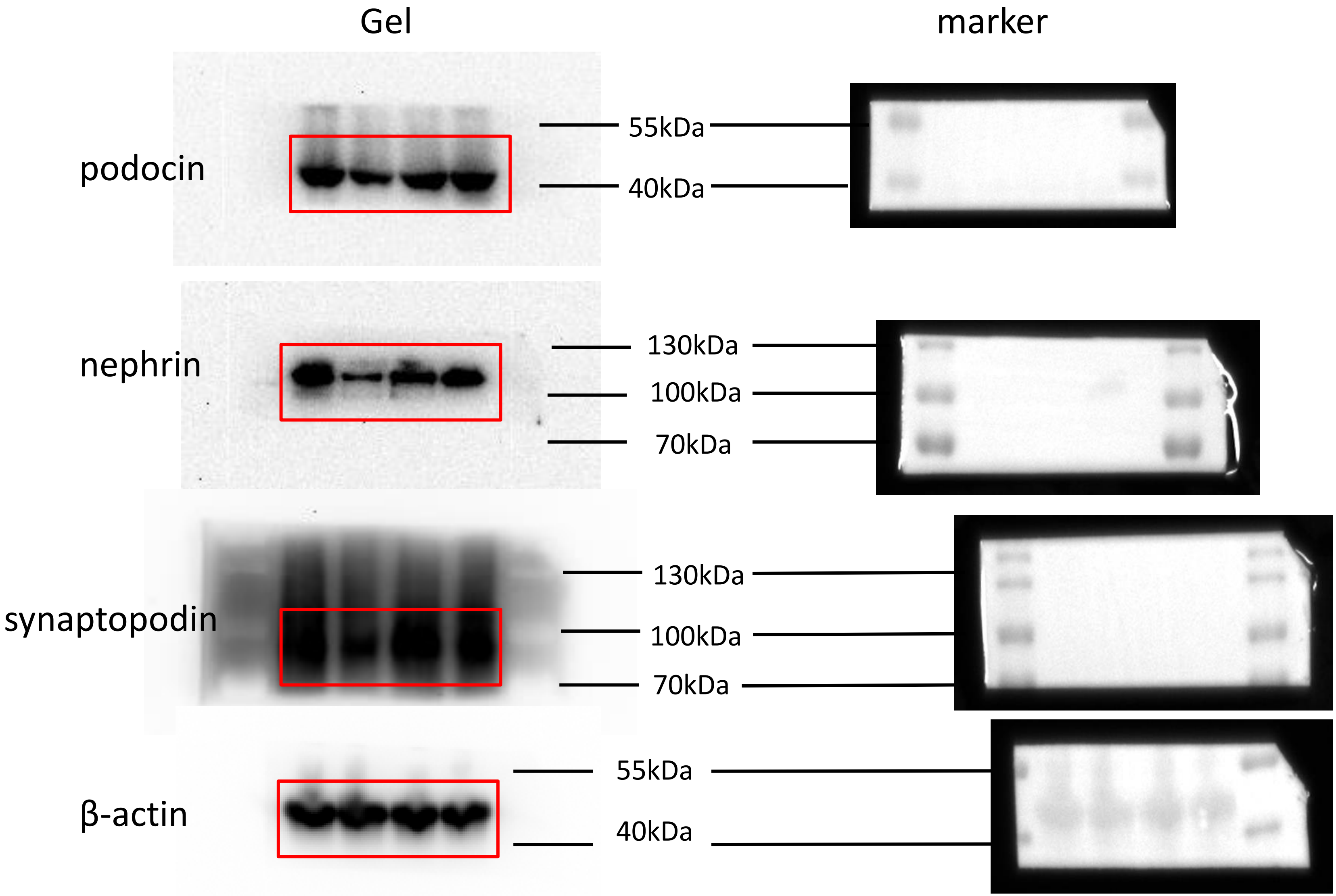

Gel
marker
podocin
55kDa
40kDa
nephrin
130kDa
100kDa
70kDa
synaptopodin
130kDa
100kDa
70kDa
β-actin
55kDa
40kDa

Figure3B

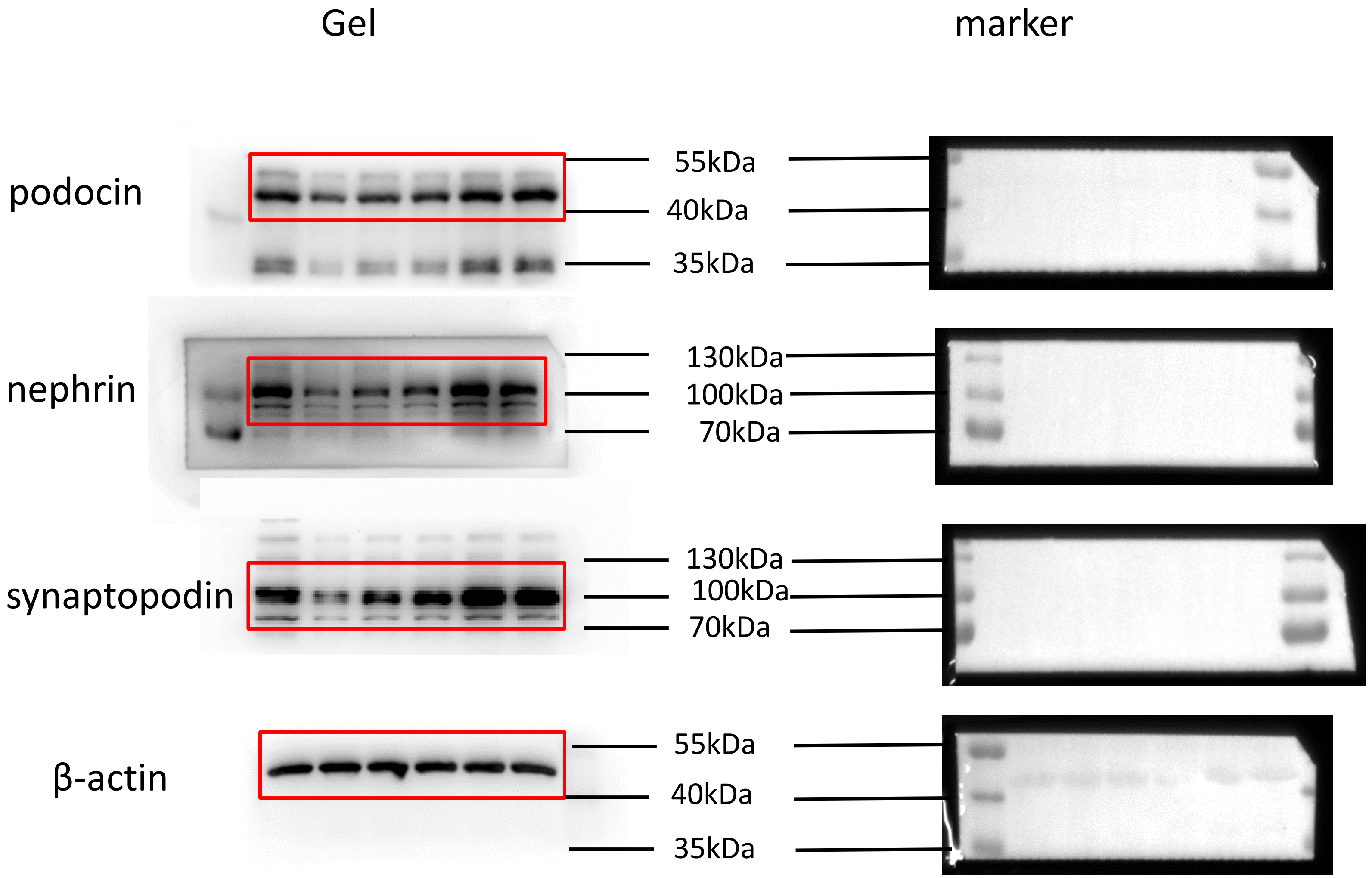
Gel
marker
podocin
55kDa
40kDa
35kDa
nephrin
130kDa
100kDa
70kDa
synaptopodin
130kDa
100kDa
70kDa
β-actin
55kDa
40kDa
35kDa

Figure4C

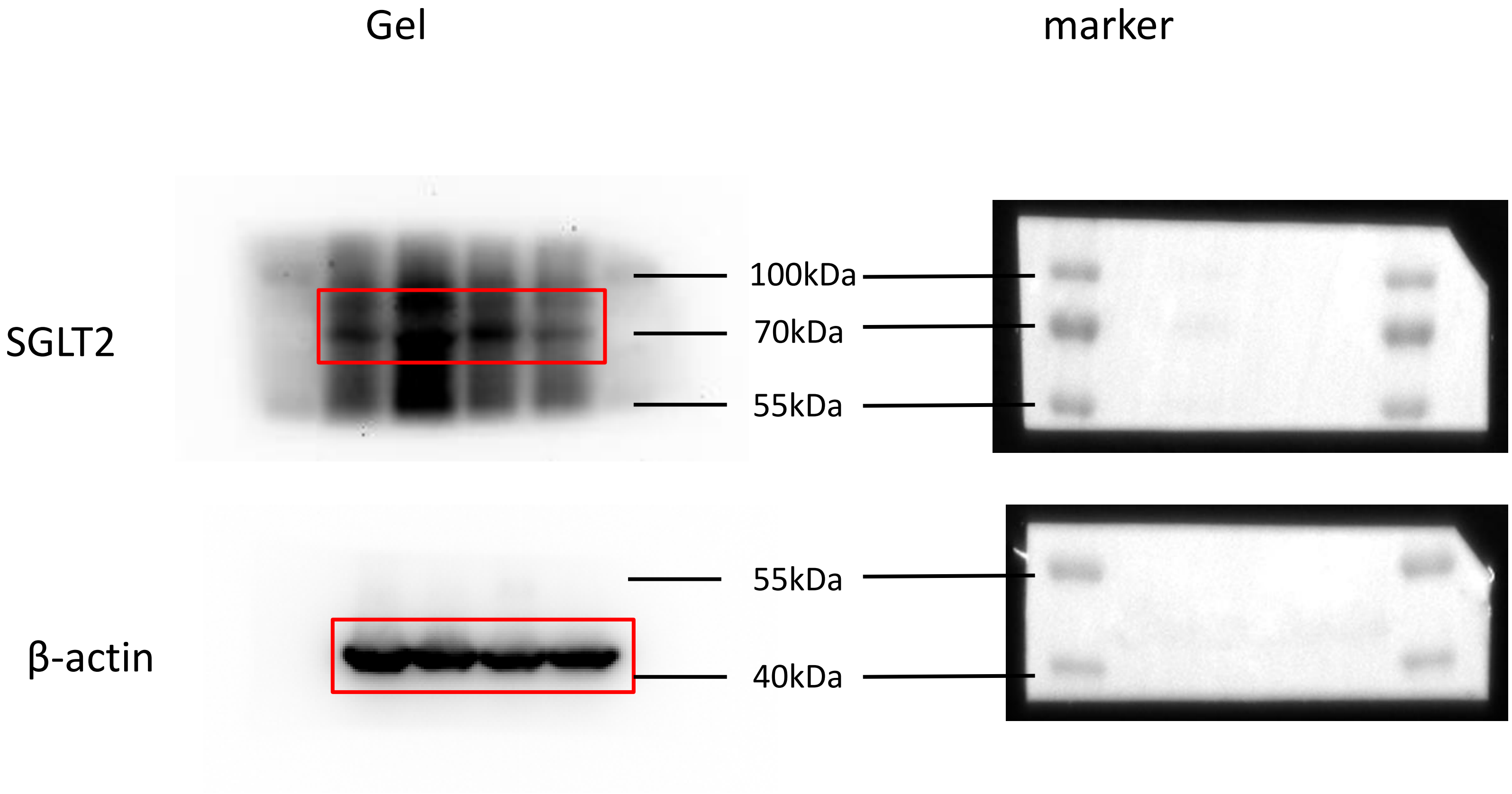

Figure 4D

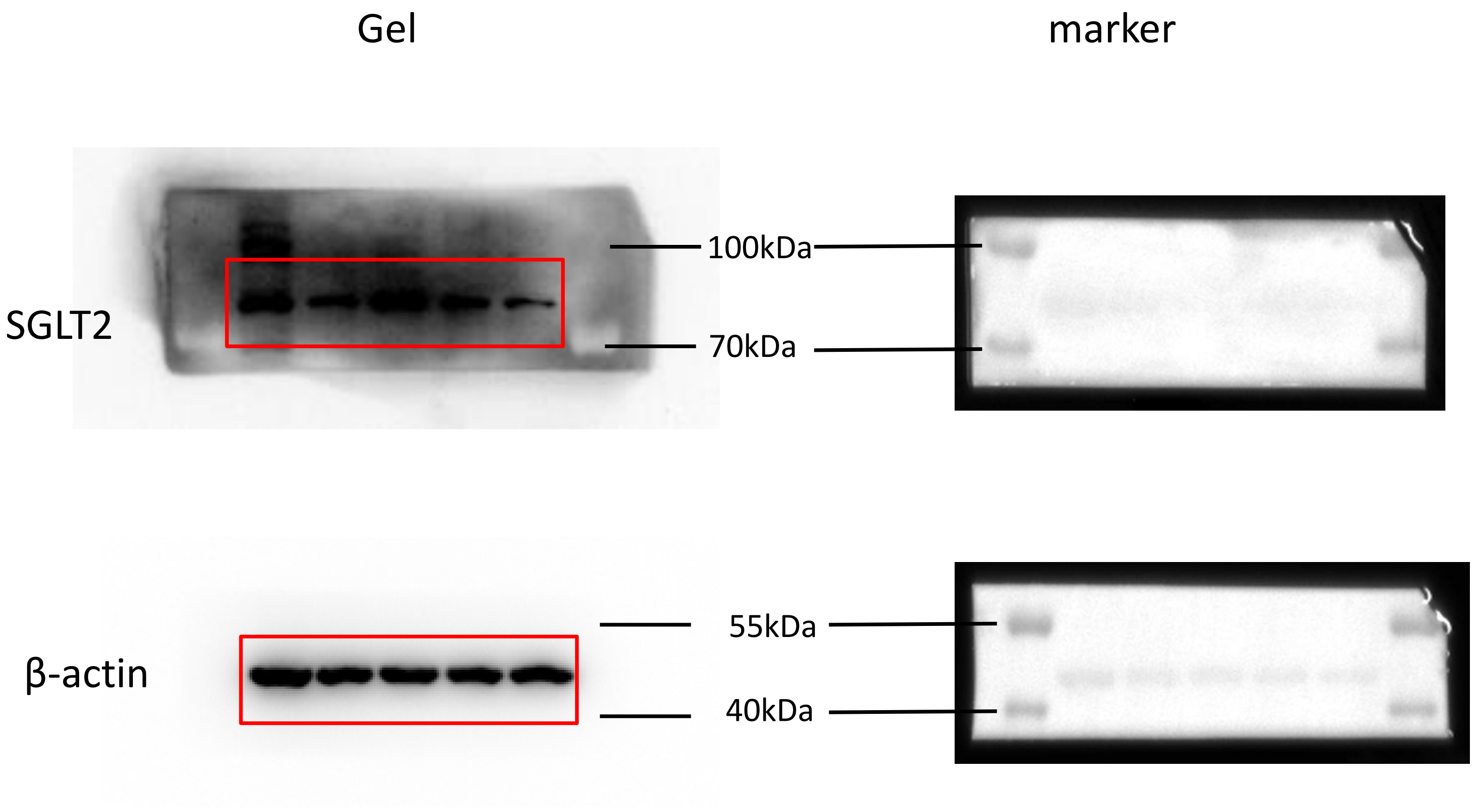

Figure5A

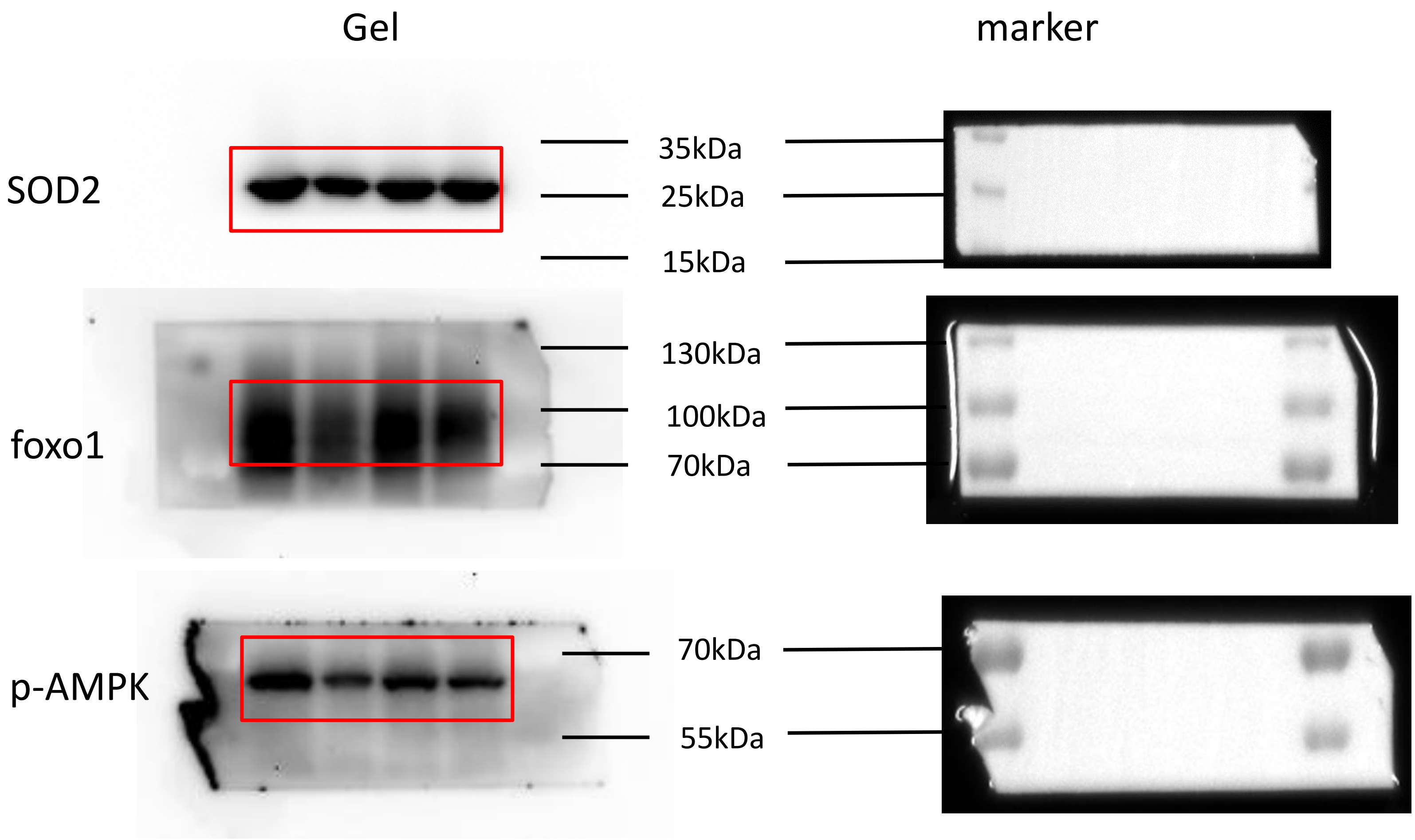
Gel
marker
SOD2
35kDa
25kDa
15kDa
foxo1
130kDa
100kDa
70kDa
p-AMPK
70kDa
55kDa

Figure5A

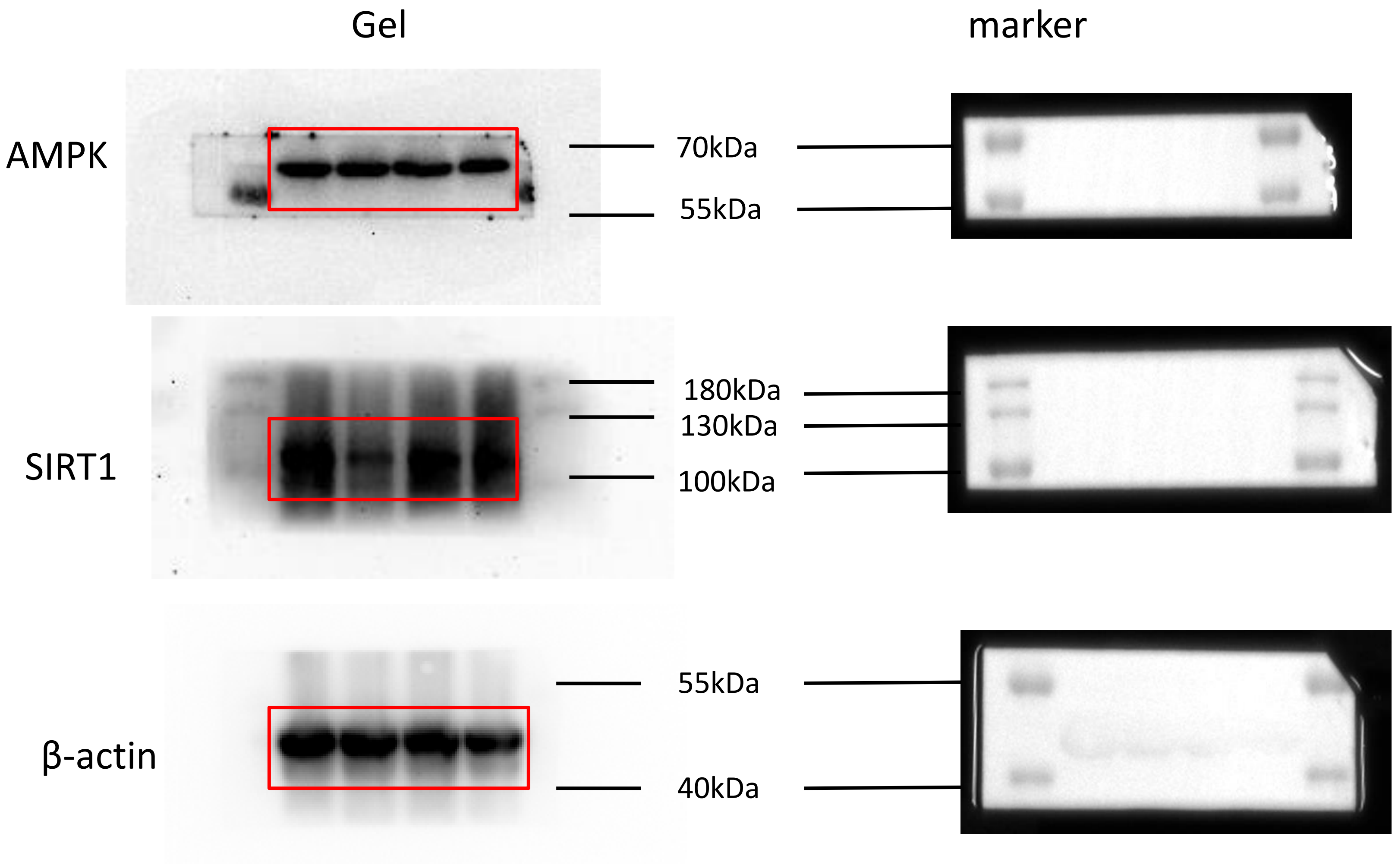
Gel
marker
AMPK
70kDa
55kDa
SIRT1
180kDa
130kDa
100kDa
β-actin
55kDa
40kDa

Figure6A

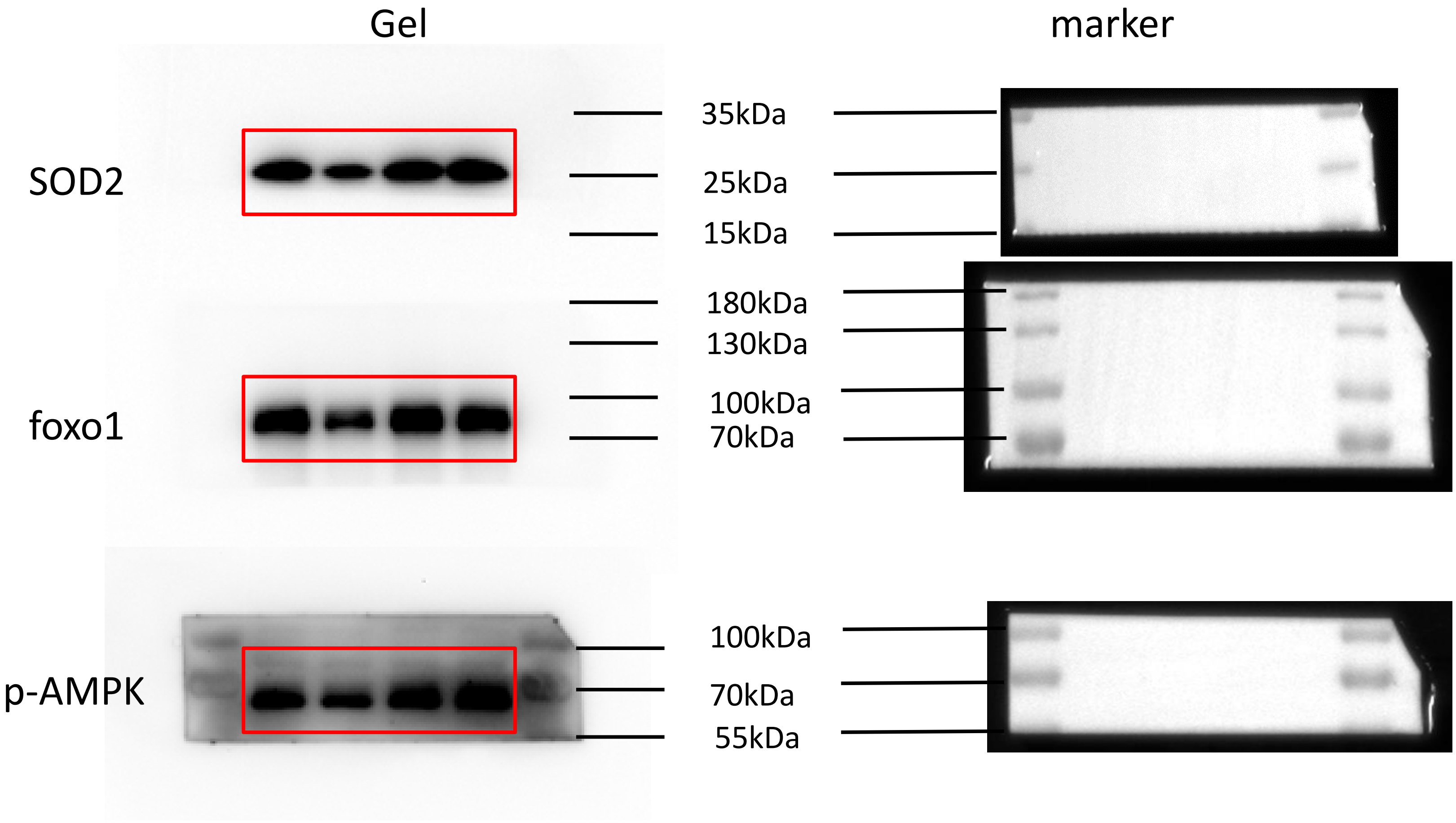
Gel
marker
SOD2
foxo1
p-AMPK
35kDa
25kDa
15kDa
180kDa
130kDa
100kDa
70kDa
100kDa
70kDa
55kDa

Figure6A

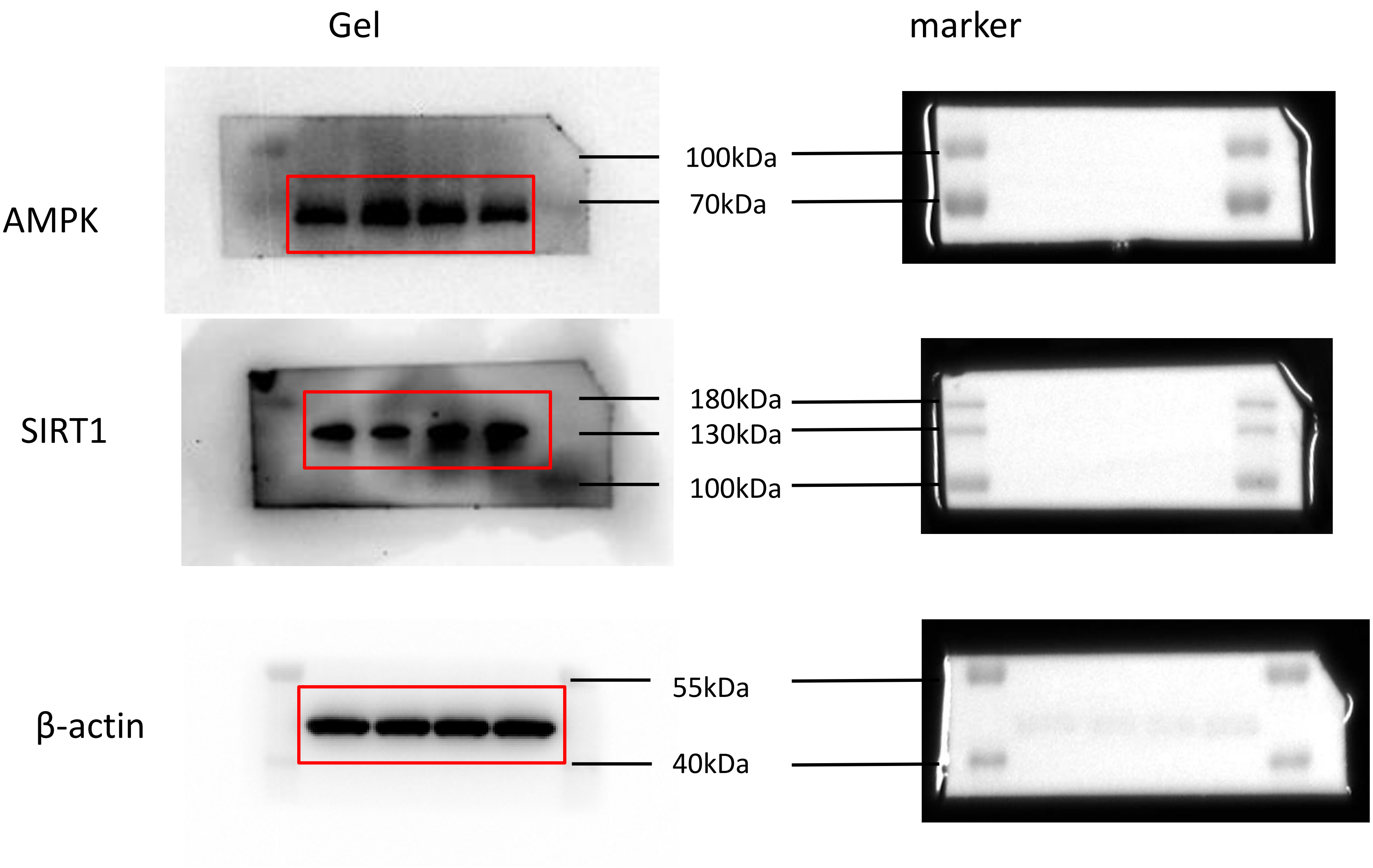
Gel
marker
AMPK
100kDa
70kDa
SIRT1
180kDa
130kDa
100kDa
β-actin
55kDa
40kDa

Figure8A

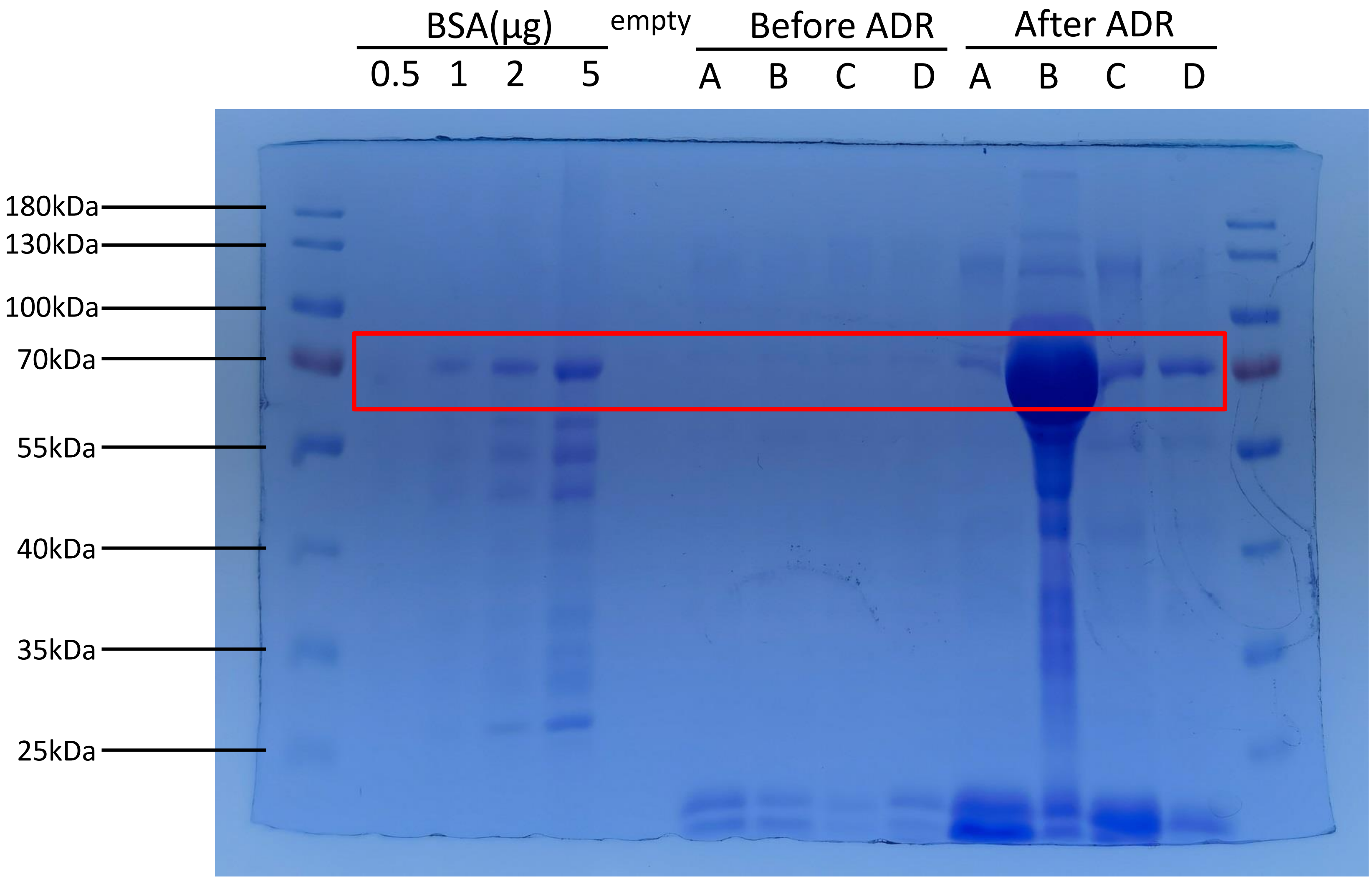

Notes： A:CTRL; B:PE; C:PE+Losartan； D： PE+EMPA
